# Low-cost, microcontroller-based phase shift measurement system for a wireless power transfer prototype

**DOI:** 10.1016/j.ohx.2022.e00311

**Published:** 2022-05-02

**Authors:** Andrés Martínez, Christian González, Adrián Jaramillo, Dorindo Cárdenas, Alejandro Von Chong

**Affiliations:** a School of Electrical Engineering, Universidad Tecnológica de Panamá, Víctor Levi Sasso Campus, Panama; b CEMCIT–AIP, SNI–SENACYT, School of Electrical Engineering, Universidad Tecnológica de Panamá, Víctor Levi Sasso Campus, Panama; c CEMCIT–AIP, School of Electrical Engineering, Universidad Tecnológica de Panamá, Víctor Levi Sasso Campus, Panama

**Keywords:** Magnetic coupling, Misalignment, Phase shift, Resonance frequency, Teensy microcontroller, Wireless power transfer

## Abstract

Seeking to characterize and mitigate the adverse effects of misalignment in WPT applications, we present the design and construction of a low-cost wireless charger prototype and a novel phase-shift measurement system. The first is built using a half-bridge inverter and antennas with series-series compensation, while a microcontroller (Teensy 4.1) supplies high-frequency PWM signals. The measurement system comprises high-speed operational amplifiers and an exclusive OR gate. A resistor was used as load. On the other hand, the maximum power transfer efficiency occurs at the resonance frequency, nevertheless, this depends physically on the geometry of the coupling system. Using a 3D-printed displacement system, we created controlled vertical misalignments between the coils, thereby obtaining variations in the resonance frequency of the system and consequently, producing a proportional phase shift between the voltage and current waves of the transmitting antenna. As the experimental results demonstrate, the measurement system can process this high-frequency signal for the phase shift estimation and subsequently use it as a control variable in a proportional-integral controller, which adjusts the operation frequency of the system and brings it back to optimal conditions. This precise yet inexpensive implementation could find its application in EVs and biomedical devices’ efficient wireless chargers.

## Graphical abstract

**Figure:**
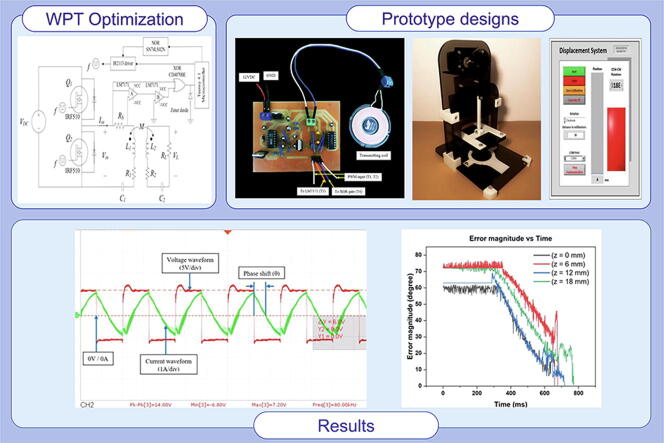

## Specifications table

**Table:** 

Hardware name	Phase shift measurement system for a wireless power transfer prototype
Subject area	• Engineering and materials science • Educational tool and open-source alternative to existing infrastructure
Hardware type	• Measuring physical properties and in-lab sensors • Field measurements and sensors • Electrical engineering and computer science
Closest commercial analog	No commercial analog is available.
Open source license	MIT License
Cost of hardware	USD 170
Source file repository	https://zenodo.org/record/6459853#.YljnKejMI2w

## Hardware in context

Wireless power transfer (henceforth referred as WPT) has become an attractive solution for charging a variety of mechanisms, such as electric vehicles, biomedical devices and cellphones. In this technology, the energy is supplied in the form of an electromagnetic wave from one or more transmitters to one or more receivers, without the use of physical connections between them [1], thus providing a convenient process for the users. However, the designs still face challenges to operate efficiently under certain circumstances. A factor that negatively impacts WPT systems is the physical misalignment between the transmitter and receiver coils during the energy transmission, whose immediate consequence of which is the decrease of the power transferred to the load.

The first documented experiments to transfer power wirelessly were carried out by Nikola Tesla in 1891 [2], who constructed a resonant circuit of loosely coupled coils to demonstrate that power could be transferred from the primary to the secondary side of the coils without conductive wires between them. Even though Tesla conceptualized the principles by which most of current inductive transfer designs operate, no safety criteria for humans or equipment were considered in his experiments, which resulted in producing very large and undesirable electric fields. Moreover, there were several limitations of applicability at the time.

Exploring more recent projects, in [3] a controller that uses both the frequency and the phase angle to optimize an inductive coupling system considering misalignment conditions was implemented. The LCCL-S topology was used, in which an inductor is placed at the inverter output as well as a filter capacitor, in addition to the series-series topology. Although the parameters of the inductive and capacitive network are provided, the switching devices used in the high frequency inverter are not detailed, nor is the device used to sense the system current mentioned. In [4] an adaptive fuzzy logic controller was developed for frequency tracking in a series-series topology WPT system. The values of the power transfer network are provided and there is an in-depth explanation of the control algorithm. However, there are no specifications for the phase shift detector implemented in the transmitting antenna or for the inverter switching devices. A characterization of the impact of misalignment on the efficiency of a WPT system was presented in [5]. Nonetheless, the inductances of the coils used are not detailed. In [6], a deep theoretical review is exposed, which uses Maxwell's equations and vector potentials to present a complete electrodynamic modeling, which has been solved using elliptical integrals and which can be used to geometrically characterize the misalignments that can exist between the transmitter and receiver coils of WPT systems.

In the research conducted, a prototype of a wireless power charger was designed and built with the objective of characterizing the effects of its operation under controlled vertical misalignments, using for this purpose, a monitoring system in the transmitting antenna. The prototype, made with series-series topology (i.e., each antenna has an inductor with a compensation capacitor connected in series), converts the power supply from a DC source into AC through a half-bridge inverter of MOSFETs, which receives the control signals from a microcontroller. The inverter powers the transmitting antenna, which wirelessly transfers the power to the receiving antenna and thus powering the load. A PI controller was used to validate the results. An evident contribution provided by this research is the complete description and specification of the materials and procedures for the construction of the WPT prototype, since in the scientific literature this information is partially provided probably due to commercial reasons. This situation hinders the elaboration of replica prototypes, but more importantly, the development of new control algorithms, novel compensation topologies as well as improvements in the power electronics. This project specifies, justifies, and explains all the materials and procedures applied, so that all the modules can be replicated without further complications. Additionally, and to our best knowledge, the phase shift measurement module is a novelty within the phase-lock loops (PLL) detection methods.

## Hardware description

The prototype’s hardware comprises four connected modules:

1. the primary-side module
2. the secondary-side module
3. the phase shift measurement module
4. the control module

### Primary-side module

**Fig. 1:**
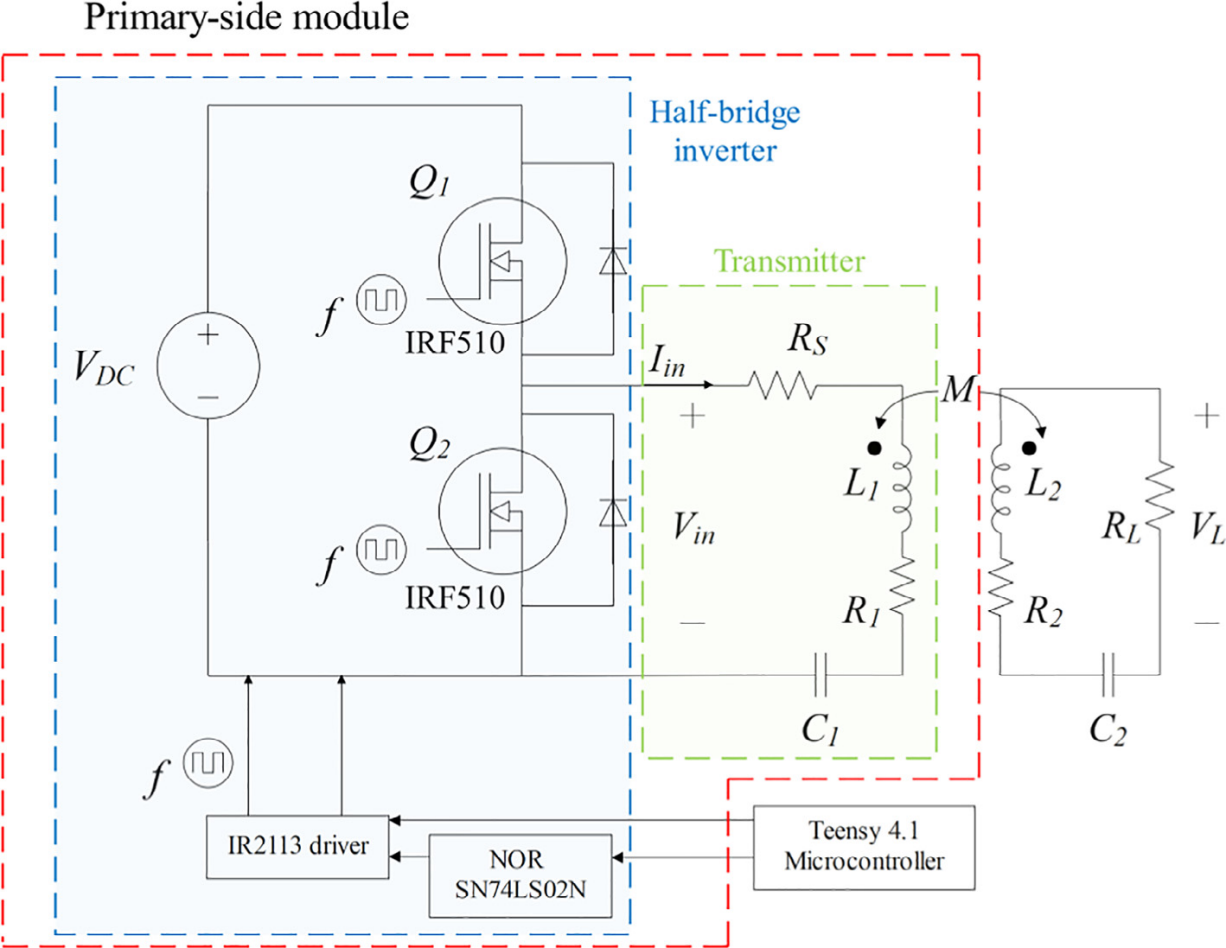
Primary-side module’s schematic.

Firstly, this module utilizes a half-bridge inverter (Fig. 1). Its main function is to transform the 12 VDC from the DC power supply (model SIGLENT SPD3303X) to a high frequency AC voltage, which is the supply for the transmitting antenna. The half-bridge inverter operates by switching two IRF510 N-channel power MOSFETs [7], whose gates are activated with an IR2113 high and low side gate driver [8]. The driver receives two PWM control signals, one directly from the Teensy 4.1 microcontroller [9] in the control module (this selection is discussed in section 2.4), and one from the NOR SN74LS02N [10] gate’s output, which is the logical complement (negation) of the first signal. Due to the characteristics of this type of inverter (half-bridge), its voltage output is a square wave that oscillates between 6 V and –6 V, and the current has a sinusoidal behavior. Since this is a low-power circuit, the use of a full-bridge inverter is not required.

The IR510 N-channel power MOSFET was selected because it has significant short rise and fall times, these being 16 and 9.4 ns [7], respectively. This is a very important characteristic since the system will be operating at frequencies in the order of kilohertz. In addition, they can switch up to 100 V, a value that is well above the 12 V we want to switch. Initially, 2 N700 MOSFETs were tested, but due to their high rise and fall times, no satisfactory results were obtained. Furthermore, the IR2113 driver was implemented to discharge the parasitic capacitances of the MOSFETs, as it amplifies the current provided by the Teensy 4.1 microcontroller and injects it into the gate of the transistors, reducing their *on* and *off* time and enhancing the system’s performance.

The second part of the primary-side module is the magnetic coupling circuit containing the transmitting antenna and its respective series-series compensation topology (Fig. 1). In the case of our system, the transmitting antenna is made up of the series connection of the coil $L_{1}$, a compensation capacitance $C_{1}$ and a resistor $R_{S}$. Additionally, $R_{1}$ is the coil internal resistance and the coefficient M represents the mutual inductance. The corresponding values are shown in the section 2.5 (Table 1).

**Table 1:** Summary of the resonant WPT system parameters. This list is crucial in the development of WPT prototypes. Unfortunately, it is not available in all projects, probably due to commercial reasons.

Parameter	Notation	Value
Input voltage (DC)	*V_DC_*	12 V
Transmitter coil self-inductance	*L_1_*	24 µH
Receiver coil self-inductance	*L_2_*	24 µH
Transmitter coil internal resistance	*R_1_*	0.07 Ω
Receiver coil internal resistance	*R_2_*	0.07 Ω
Transmitting antenna compensation capacitor	*C_1_*	200 nF
Receiving antenna compensation capacitor	*C_2_*	200 nF
Shunt resistor	*R_S_*	3 Ω
Load resistor	*R_L_*	100, 500, 1000 Ω
Operating frequency	*f*	50 – 90 kHz

**Fig. 2:**
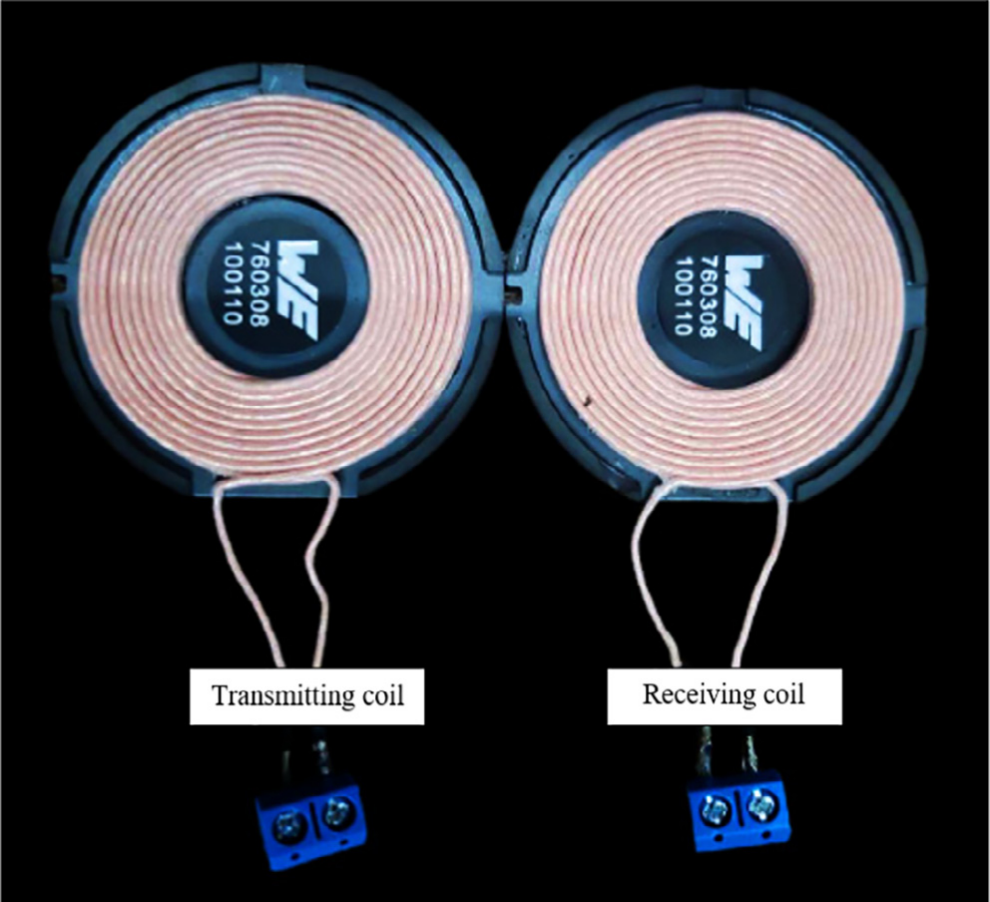
Transmitter and receiver coils from the manufacturer Würth Elektronik.

The coils (Fig. 2), manufactured by Würth Elektronik (Hohenlohe, Germany), are a special product for wireless charging applications, as they comply with the Qi standard of the Wireless Power Consortium [11]. Unlike other commercial solutions, their winding is made of Litz wire, a multi-stranded conductor covered by an insulating film that is particularly used in radio frequencies to reduce losses due to the skin and proximity effects. Additionally, the winding rests on a material with high magnetic permeability (ZnNi), which increases the coupling factor and consequently, the power transfer. The inductance of both coils is 24 ± 10% µH, as specified in (Table 1).

The compensation capacitance of both networks is 200 nF, as specified in (Table 1). In both antennas we use ceramic capacitors. We intended them to compensate for the parasitic and inherent capacitance of the coils. Ceramic capacitors have lower equivalent series resistance and equivalent series inductance. As a result, they perform better against transients than electrolytic capacitors [12]. This behavior was corroborated through laboratory tests, in which the electrolytic capacitors could not stand sudden operating frequency changes at high frequencies.

It is important to highlight that WPT systems generally operate at high frequencies (hundreds of kilohertz). This is because the efficiency of the magnetic coupling is directly proportional to the quality factors of the coils, which in turn are proportional to the frequency [13]. Furthermore, a desirable condition is to operate at the resonance frequency since the transferred power is maximized [1], hence this frequency must be calculated. For practical purposes, the approximation proposed in [1], [14] will be applied. In both approaches, it is suggested to consider the two systems as independent LC oscillators which resonate at the same frequency; thus, giving as a theoretical yet approximate solution the following expression: $f=1/(2\pi \sqrt{\mathrm{LC}})$. This yields an approximate resonance frequency of 72.6 kHz for the proposed system. This value is appropriate for EVs charging, as it is included in the 10 – 150 kHz range described in [15], and it is also close to the one recommended by the SAE J2954 standard of 79 – 90 kHz [16]. On the other hand, the considered frequency of 72.6 kHz is approved for active implantable medical device (AIMD) applications, according to [17], [18]. Furthermore, recently developed WPT implementations in AIMD successfully operate in a wide range; specifically, at 20 kHz [19], 300 kHz and 13.56 MHz [20].

### Secondary-side module

**Fig. 3:**
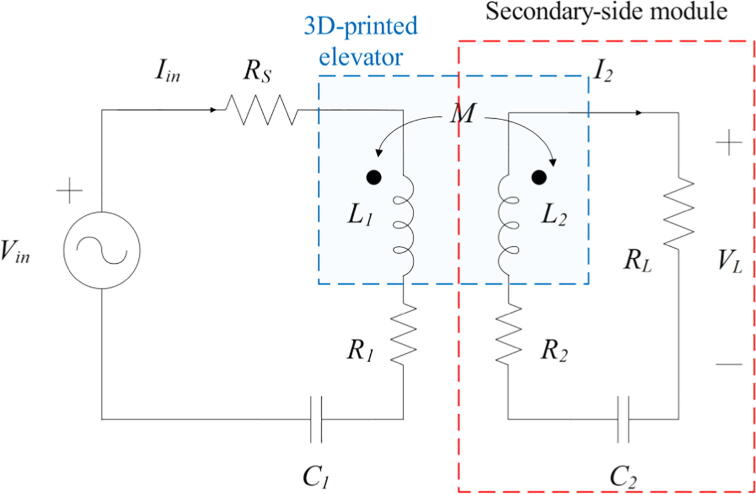
Secondary-side module’s schematic.

The secondary-side module (Fig. 3) is constituted by the receiving coil and its compensation network, and the load resistor. First, the receiving antenna is composed by the series connection between $L_{2}$, the compensation capacitance $C_{2}$ and the resistor $R_{L}$, which represents the load. In addition, $R_{2}$ is the coil internal resistance and the coefficient M represents the mutual inductance. The corresponding values are shown in the assembly section (Table 1).

Note that the transmitting and receiving coils are fixed at the bottom and top of the 3D-printed displacement system, respectively. The build instructions section gives more details about the displacement system.

### Phase shift measurement module

Besides the complete and unrestricted hardware description, another important contribution of this project is the phase shift measurement module, whose theoretical background is discussed in this section.

In the resonance condition (optimal operating condition), the absence of imaginary component in the input impedance means that it has zero phase angle. Accordingly, this implies that the voltage and current waves in the transmitting antenna are in phase when the system resonates. Hence, the phase shift measurement module takes the measurements from the transmitting antenna and its purpose is to serve as an indicator of the resonance condition.

**Fig. 4:**
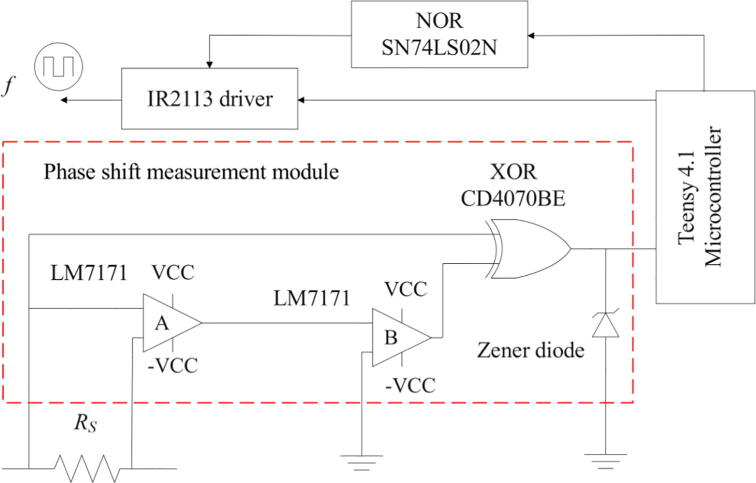
Phase shift measurement module’s schematic.

For measuring the phase shift, we propose a system that comprises two LM7171 operational amplifiers [21], special for high-speed operation (4100 V/µs of slew rate), and an exclusive-OR (XOR) gate model CD4070BE [22] (Fig. 4). For reference, the slew rate of a conventional operational amplifier (LM741) is 0.5 V/µs [23], i.e., approximately 8000 times slower.

Firstly, the LM7171 ‘A’ samples the sinusoidal current wave in the $R_{S}$ resistor and amplifies it to an appropriate voltage level. Secondly, the LM7171 ‘B’ transforms this amplified sinusoidal wave into a square wave and sends it to one of the inputs of the XOR gate. On the other hand, the voltage wave of the primary-side module is sent directly to other input of the XOR gate. The latter compares both signals and generates a pulse whose width indicates the number of seconds that the current wave leads or lags the voltage wave. In other words, the measurement system compares the zero-crossing (i.e., the point where the sign of the function changes) of the signals and indicates the time difference between them by generating a pulse. The pulse’s width (*on* time or *off* time) and frequency are read by the Teensy 4.1 board in microseconds and kHz, respectively. Subsequently, the microcontroller’s algorithm computes the phase angle according to section 2.4. A Zener diode between the XOR gate’s output and the Teensy 4.1 is necessary to reduce the original voltage level of the pulse to one of logic level, which the microcontroller can withstand.

### Control module

**Fig. 5:**
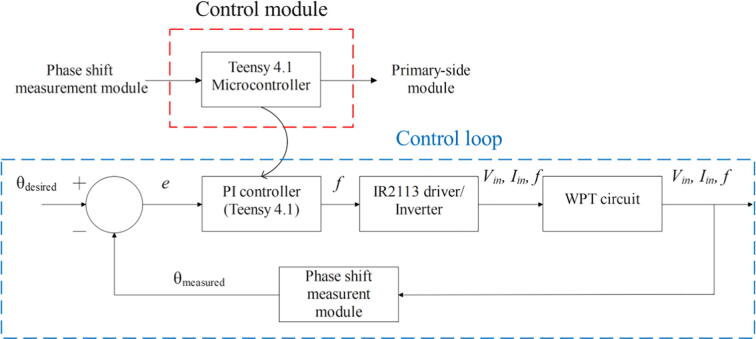
Control module’s schematic.

Fig. 5 shows the control module’s schematic. Teensy 4.1 was selected as the microcontroller for the control module because it offers the same programing environments as an Arduino board, but with a 600 MHz processor [9], which is approximately 35 times faster than the 16 MHz one of the Arduino ATmega328p [24]. The coding was developed in VS code, a broadly used environment by Arduino’s owners due to its intuitive interfaces and ease of use. Besides, one can keep using the same programing language (Arduino), which is already well documented and has plenty of useful libraries. Furthermore, Teensy 4.1 only consumes 100 mA and provides support for dynamic clock scaling. To be specific, unlike traditional microcontrollers, Teensy 4.1 board and Teensyduino's software allow dynamic speed changes in their timing functions, whereas other similar boards present issues under these circumstances, such as erroneous baud rates.

The control module performs a variety of tasks:

• Provide the PWM control signal to the primary-side module.
• Receive and read the pulse’s width and frequency from the phase shift measurement module.
• Calculate the phase angle between the voltage and current waves of the transmitting antenna.
• Compute the new operating frequency by means of a PI controller. This controller requires a prior selection of the proportional and integral gains (kp and ki, respectively).

Firstly, the microcontroller takes several samples (specify by the operator) of the pulse’s width (Td) and frequency (f) and then calculates an average measurement. This data is used in the equation phase angle[°] = 360° * f[Hz]*Td[s] to compute the phase shift between the waves. Secondly, the PI controller algorithm calculates the error with respect to a desired angle (zero degrees for this application, which corresponds to the resonance frequency) and then performs the product of the error times the proportional gain ($k_{p}$) and the sum of errors times the integral gain ($k_{i}$). Next, these two terms are added to obtain the new operating frequency. This process is iterated until the resonance condition is reached (i.e., until the frequency calculated produces zero phase shift and thus, the power transferred is maximized).

The corresponding coding files for developing all the tasks are specified in the design files summary and explained in the build instructions section. In addition, illustrations of the measurement system results are shown in the validation and characterization section.

### Modules assembly

**Fig. 6:**
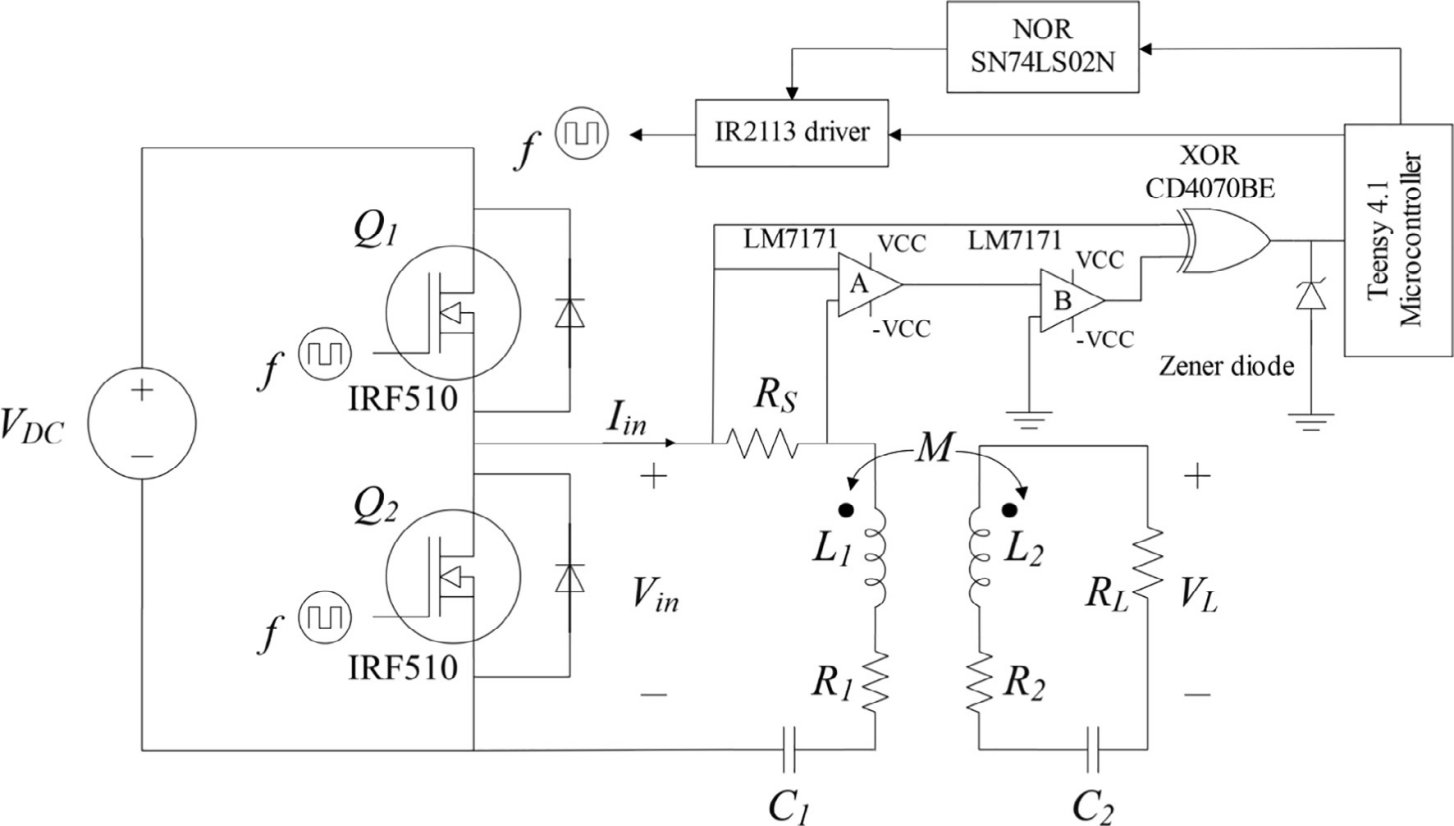
Schematic of the four modules assembled.

After connecting all the four modules described in the past sections, the resultant assembly contains both the WPT prototype and the phase shift measurement system (Fig. 6). The values and specifications of the parameters observed in the schematic are listed in Table 1.

## Design files summary

The wiring diagrams, as well as the PCB layouts and Gerber files for the PCB manufacturing were generated using the design software EasyEDA, which can load the JSON files listed in Table 2.Table 3..

**Table 2:** Design files list. All files are available in the online repository: https://zenodo.org/record/6459853#.YljnKejMI2w.

Design file name	File type	Open source license
Primary_side_wiring_diagram	JSON file; PDF file	MIT license
Primary_side_PCB_layout	JSON file; PDF file	MIT license
Primary_side_PCB_manufacturing	ZIP file	MIT license
Secondary_side_wiring_diagram	JSON file; PDF file	MIT license
Secondary_side_PCB_layout	JSON file; PDF file	MIT license
Secondary_side_PCB_manufacturing	ZIP file	MIT license
Phase_shift_wiring_diagram	JSON file; PDF file	MIT license
Phase_shift_PCB_layout	JSON file; PDF file	MIT license
Phase_shift_PCB_manufacturing	ZIP file	MIT license
Control_module_PCB_layout	JSON file; PDF file	MIT license
Control_module_PCB_manufacturing	ZIP file	MIT license
PWM_signals_test	INO file	MIT license
Pulse_width_frequency	INO file	MIT license
pi_controller	INO file	MIT license
DS_instructions_drawings	PDF file	MIT license
DS_wiring_diagram	JSON file; PDF file	MIT license
DS_PCB_layout	JSON file; PDF file	MIT license
DS_PCB_manufacturing	ZIP file	MIT license
DS_modifiable_3D_design	F3D file	MIT license
3D_printable_support_structures	ZIP file	MIT license
Acrylics_laser_cutting	ZIP file	MIT license
DS_assembly_views	ZIP file	MIT license
DS_coding	ZIP file	MIT license
Rot-Tras_Mechanism	INO file	MIT license
LabVIEW_interface_Rot-trans	ZIP file	MIT license
AcquisitonSoftware	VI file	MIT license

**Table 3:** Bill of materials.

Designator	Component	Number	Cost per unit – USD	Total cost – USD	Source of materials	Material type
L1, L2	Wireless charging coil, WE-WPCC Series (24 µH ± 10%)	2	16.29	32.58	Newark	Litz wire, ZiMn
C1A, C1B, C2A, C2B	Multilayer ceramic capacitors leaded (50 V, 100nF, 10%)	4	0.51	2.04	Mouser Electronics	Ceramic
C3, C4, C6, C7, C9, C10	Aluminum Electrolytic Capacitor – Radial Leaded 0.1uF 100 V	6	0.28	1.68	Mouser Electronics	Aluminum
C5	Aluminum Electrolytic Capacitor – Radial Leaded 100 V 0.22uF	1	0.27	0.27	Mouser Electronics	Aluminum
C8	Aluminum Electrolytic Capacitor – Radial Leaded 25 V 10uF	1	0.27	0.27	Mouser Electronics	Aluminum
C11	Multilayer Ceramic Capacitor MLCC – Leaded 50 V 10pF	1	0.44	0.44	Mouser Electronics	Ceramic
C12	Aluminum Electrolytic Capacitors – Radial Leaded	1	0.63	0.63	Mouser Electronics	Aluminum
R_L_	Carbon Film Resistors – Through Hole 100Ohm 2 W 500PPM	1	0.067	0.067	Mouser Electronics	Carbon film
R_L_	510-ohm Resistor 1/2w (0.5Watt) ± 1% Tolerance Metal Film Fixed Resistor	1	0.60	0.60	Amazon	Metal film
R_L_	Carbon Film Resistors – Through Hole 1 K Ohm 1 W 5% 500 Volt	1	0.33	0.33	Mouser Electronics	Carbon film
R3, R4	Carbon Film Resistor – Through Hole 22 Ohm 1/4W 5% 250 V	2	0.13	0.26	Mouser Electronics	Carbon film
R5, R6, R8, R10	Carbon Film Resistor – Through Hole 10 K Ohm 1/4W 5% 250 V	4	0.13	0.52	Mouser Electronics	Carbon film
R7, R9, R12	Carbon Film Resistor – Through Hole 4.7 K Ohm 5%	3	0.10	0.3	Mouser Electronics	Carbon film
R11, R13	Wirewound Resistor – Through Hole 3 W 52 O 1%	2	2.02	4.04	Mouser Electronics	Wirewound
Rs	100 pcs 3-ohm Resistor 1/4 W ± 1% Metal Film Fixed Resistor	1	0.50	0.50	Amazon	Metal film
Q1, Q2	N-channel power MOSFET (100 V, 5.6 A)	2	1.04	2.08	Mouser Electronics	Semiconductor
IR2113 driver	IR2113 Hi&Lw side MOSFET gate driver	1	4.86	4.86	Mouser Electronics	Semiconductor
NOR gate	SN74LS02N Quad 2-imput logic gate	1	0.82	0.82	Mouser Electronics	Semiconductor
Teensy 4.1	ARM Teensy 4.1 development board	2	29.38	58.76	Mouser Electronics	Non-specific
LM7805	LM7805CT 5 V linear voltage regulator	1	1.96	1.96	Mouser Electronics	Semiconductor
XOR gate	XOR CD4070BE logic gate quad exclusive	1	0.69	0.69	Mouser Electronics	Semiconductor
LM7171 Op. Amp.	LM7171BIN Op Amps Hi-Spd Hi-Output	2	4.31	8.62	Mouser Electronics	Semiconductor
D1	1 N4007-T diode (1 V, 1A)	1	0.41	0.41	Mouser Electronics	Semiconductor
D2	1 N5225 Zener diode (3.0 V, 0.5 W, 5%)	1	0.19	0.19	Mouser Electronics	Semiconductor
Fixed terminal blocks	Fixed Terminal Blocks 2 24 Poles, Screw Type, 5.0 Pitch	11	0.66	7.26	Mouser Electronics	Non-specific
PCB copper clad	10× single-sided copper clad (100×70×1.5 mm)	1	16.11	16.11	Amazon	Copper
Plastic acrylic sheets	Clear acrylic plexiglas sheet, pack of 2 (8×12×1/4 in)	1	6.51	6.51	Amazon	Acrylic
Breadboard jumper wires	120× Multicolored MM, MF, FF Breadboard Jumper Wires	1	6.98	6.98	Amazon	Non-specific
Stepper motor	Twotrees Stepper Motor Nema 17 Motor High Torque 1.5A	1	9.99	9.99	Amazon	Non-specific
Stepper motor driver	2Pack A4988 Stepper Motor Driver Module	1	1	1.00	Electronicscomp	Non-specific
**TOTAL**				**170.17**		

Note: the cost of the 3D-printed pieces was not included in this bill of materials.

The coding files (INO files) were developed in the VS code programing environment. Note that this environment requires the installation of the Teensyduino and VisualTeensy add-ons, as well as the Arduino extension to work along properly with the Teensy 4.1 board. Alternatively, regarding this application, the Arduino IDE also works perfectly, and it only needs the Teensyduino add-on. More detailed information about the programing is provided in the build instructions section and the operation instructions section.

The 3D models were produced with the software Autodesk Fusion 360. All the STL files are located inside 3D_printable_support_structures.zip and the modifiable version in DS_modifiable_3D_design.f3d. On the other hand, the files for the laser cutting can be found in Acrylics_laser_cutting.zip. For the displacement system assembly instructions, refer to section 5.5.

**Primary_side_wiring_diagram:** detailed electrical connections of the primary-side module (JSON file can be loaded into EasyEDA directly).

**Primary_side_PCB_layout:** layout of the primary-side module’s PCB (JSON file can be loaded into EasyEDA directly).

**Primary_side_PCB_manufacturing:** files required for manufacturing the primary-side module’s PCB (GTL, GBL, DRL).

**Secondary_side_wiring_diagram:** detailed electrical connections of the secondary-side module (JSON file can be loaded into EasyEDA directly).

**Secondary_side_PCB_layout:** layout of the secondary-side module’s PCB (JSON file can be loaded into EasyEDA directly).

**Secondary_side_PCB_manufacturing.:** files required for manufacturing the secondary-side module’s PCB (GTL, GBL, DRL).

**Phase_shift_wiring_diagram:** detailed electrical connections of the phase shift measurement module (JSON file can be loaded into EasyEDA directly).

**Phase_shift_PCB_layout:** layout of the phase shift measurement module’s PCB (JSON file can be loaded into EasyEDA directly).

**Phase_shift_PCB_manufacturing:** files required for manufacturing the phase shift measurement module’s PCB (GTL, GBL, DRL).

**Control_module_PCB_layout:** layout of the control module’s PCB (JSON file can be loaded into EasyEDA directly).

**Control_module_PCB_manufacturing:** files required for manufacturing the control module’s PCB (GTL, GBL, DRL).

**PWM_signals_test:** code for the basic, initial test of the prototype.

**Pulse_width_frequency:** code for measuring in the Teensy 4.1 board the width and the frequency of the pulse, thus calculating the phase angle.

**pi_controller:** code for optimizing the system by means of the PI controller.

**DS_instructions_drawings:** step by step assembly instructions for the displacement system (DS) and details of every component.

**DS_wiring_diagram:** detailed electrical connections of displacement system (JSON file can be loaded into EasyEDA directly).

**DS_PCB_layout:** layout of the displacement system’s PCB (JSON file can be loaded into EasyEDA directly).

**DS_PCB_manufacturing:** files required for manufacturing the displacement system’s PCB (GTL, GBL, DRL).

**DS_modifiable_3D_design:** F3D file (compatible with Autodesk Fusion360) that contains the complete 3D design of the displacement system.

**3D_printable_support_structures:** contains all the STL files for the 3D printing.

**Acrylics_laser_cutting:** contains all the drawings for the laser cutting in DXF format.

**DS_assembly_views:** contains images (isometric view, top view, back view, front view) of the final assembly of the displacement system.

**DS_coding:** folder containing the coding and the libraries for operating the displacement system.

**Rot-Tras_Mechanism:** code for operating the vertical displacement system along with the LabVIEW interface.

**LabVIEW_interface_Rot-trans:** folder containing all the files required to utilize the displacement system’s interface.

**AcquisitonSoftware:** file containing the data acquisition software developed in LabVIEW.

## Bill of materials summary

Additionally, a list of supplementary devices that were used in the manufacturing process is provided in Table 4. These devices are commonly found in electrical engineering or fabrication laboratories. However, if you do not have access to similar devices, we strongly recommend buying the.

**Table 4:** Bill of supplementary materials.

Designator	Component	Number	Cost per unit – USD	Total cost – USD	Source of materials	Material type
V_DC_	SIGLENT SPD3303X DC Power Supply	1	539.00	539.00	Amazon	Non-specific
Oscilloscope	SIGLENT SDS1104X-E 100Mhz digital oscilloscope 4 channels	1	499.00	499.00	Amazon	Non-specific
Function generator	SIGLENT SDG1032X Arbitrary Waveform Function Generator	1	319.00	319.00	Amazon	Non-specific
3D printer	R QIDI TECHNOLOGY 3D Printer High Precision Printing (10.6x7.9x7.9 in)	1	699.00	699.00	Amazon	Non-specific
Laser-cutting machine	Glowforge Basic 3D laser printer	1	2995.00	2995.00	Glowforge	Non-specific
Milling machine	Bantam Tools desktop CNC milling machine	1	3999.00	3999.00	Bantam tools	Non-specific
Soldering station	Soldering Iron Kit Sookey 80 W Fast Ceramic Heating Soldering Gun	1	15.99	15.99	Amazon	Non-specific

## Build instructions

### Primary-side module manufacturing

**Fig. 7:**
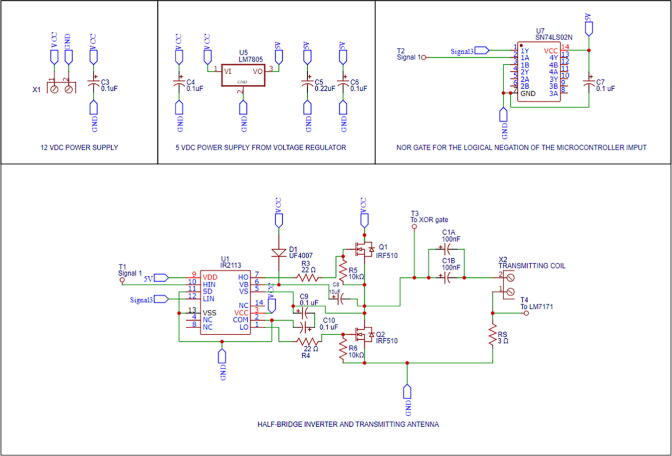
Primary-side module’s wiring diagram.

**Fig. 8:**
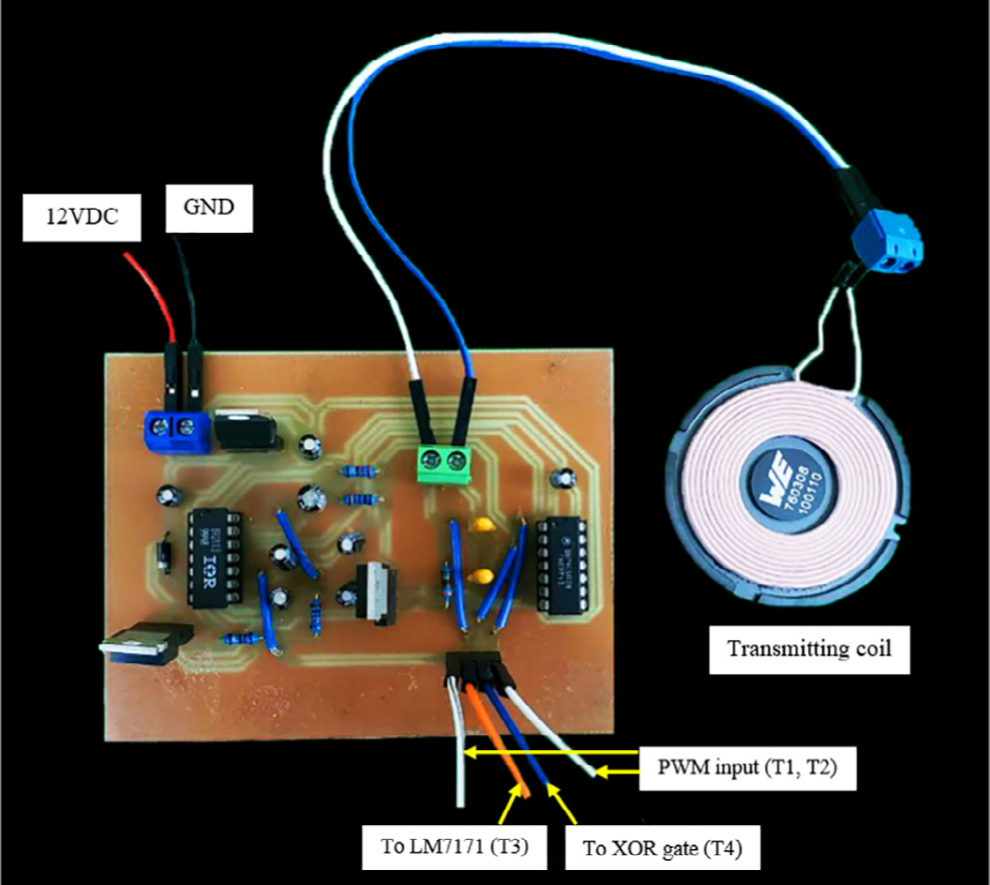
Primary-side module’s PCB.

Fig. 7 illustrates the wiring diagram for the first module (also found in Primary_side_wiring_diagram.pdf). These connections can be made in a breadboard, although this option is strongly discouraged due to the high-frequency signals and high-speed components present in the circuit. The best procedure then is to manufacture a printed circuit board (PCB) using a design software, such as EasyEDA, and use a milling machine to print it in a copper clad (100×70 mm size is adequate). The circuit’s diagram can be directly opened with any design software by uploading the file Primary_side_wiring_diagram.json. Subsequently, the Gerber files necessary for the PCB printing can be generated from the diagram. Our PCB layout is available in Primary_side_PCB_layout.json. In addition, the files required by the milling machine software can be found in Primary_side_PCB_manufacturing.zip. We recommend installing a 0.8 mm bit for cutting the traces and drilling the holes. The primary-side module PCB assembly is shown in Fig. 8.

Note that after placing the components, they must be soldered carefully to their corresponding pads with solder wire. Due to the high temperatures reached by the soldering iron’s tip, caution is required. Check with a digital multimeter the traces’ continuity and remove any undesired short circuits before the operation.

### Secondary-side module manufacturing

**Fig. 9:**
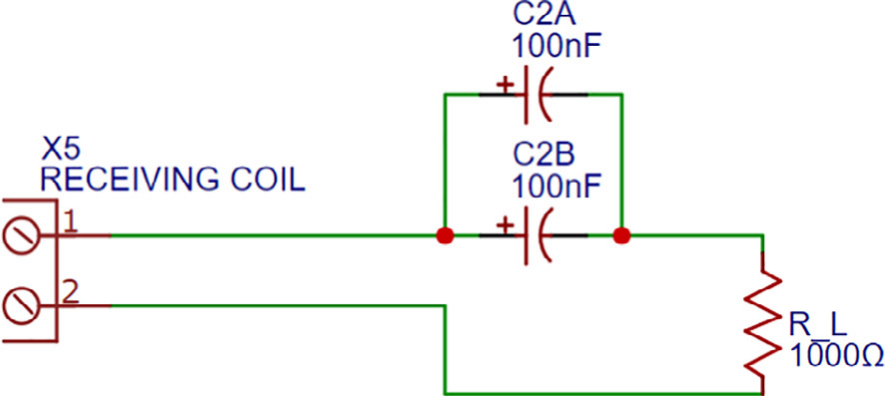
Secondary-side module’s wiring diagram.

**Fig. 10:**
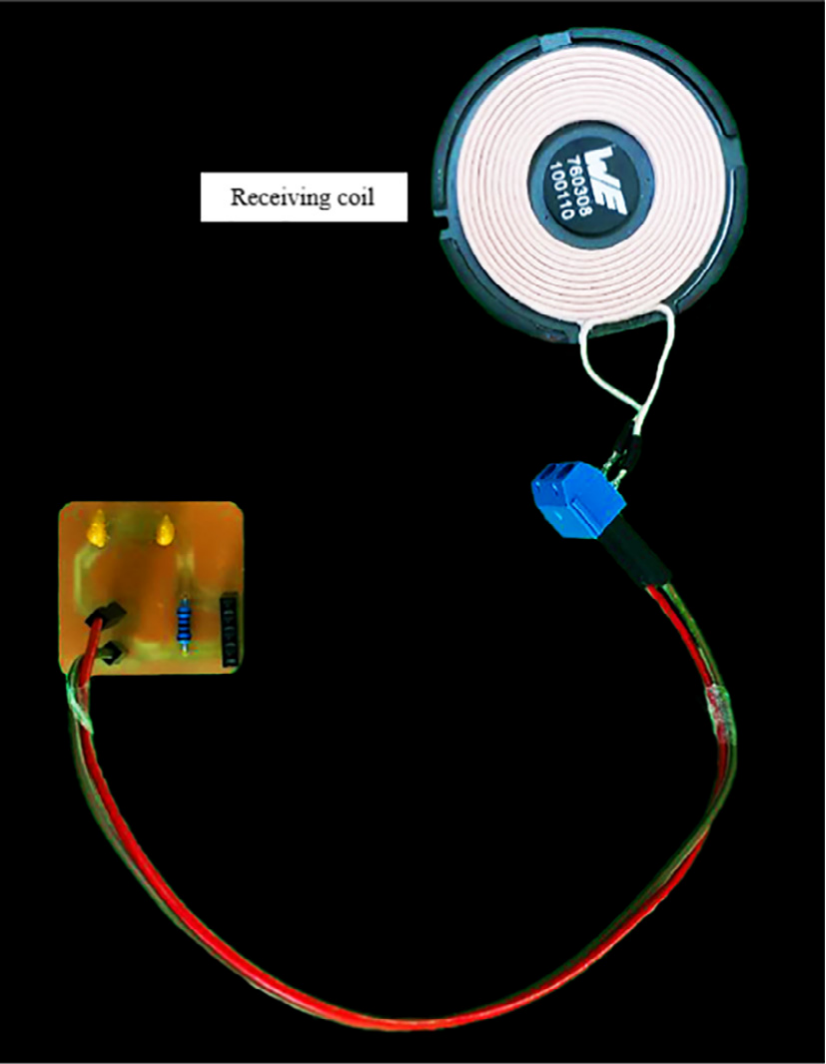
Secondary-side module’s PCB.

The secondary-side module’s wiring diagram is shown in Fig. 9, which is also available in Secondary_side_wiring_diagram.pdf. The PCB layout and the PCB manufacturing files are found in Secondary_side_PCB_layout.json and Secondary_side_PCB_manufacturing.zip, respectively. Fig. 10 illustrates the secondary-side module’s PCB assembly. Similarly to the previous section, follow the same instructions for manufacturing the PCB. The same safety concerns and recommendations are applied to this module.

### Phase shift measurement module manufacturing

**Fig. 11:**
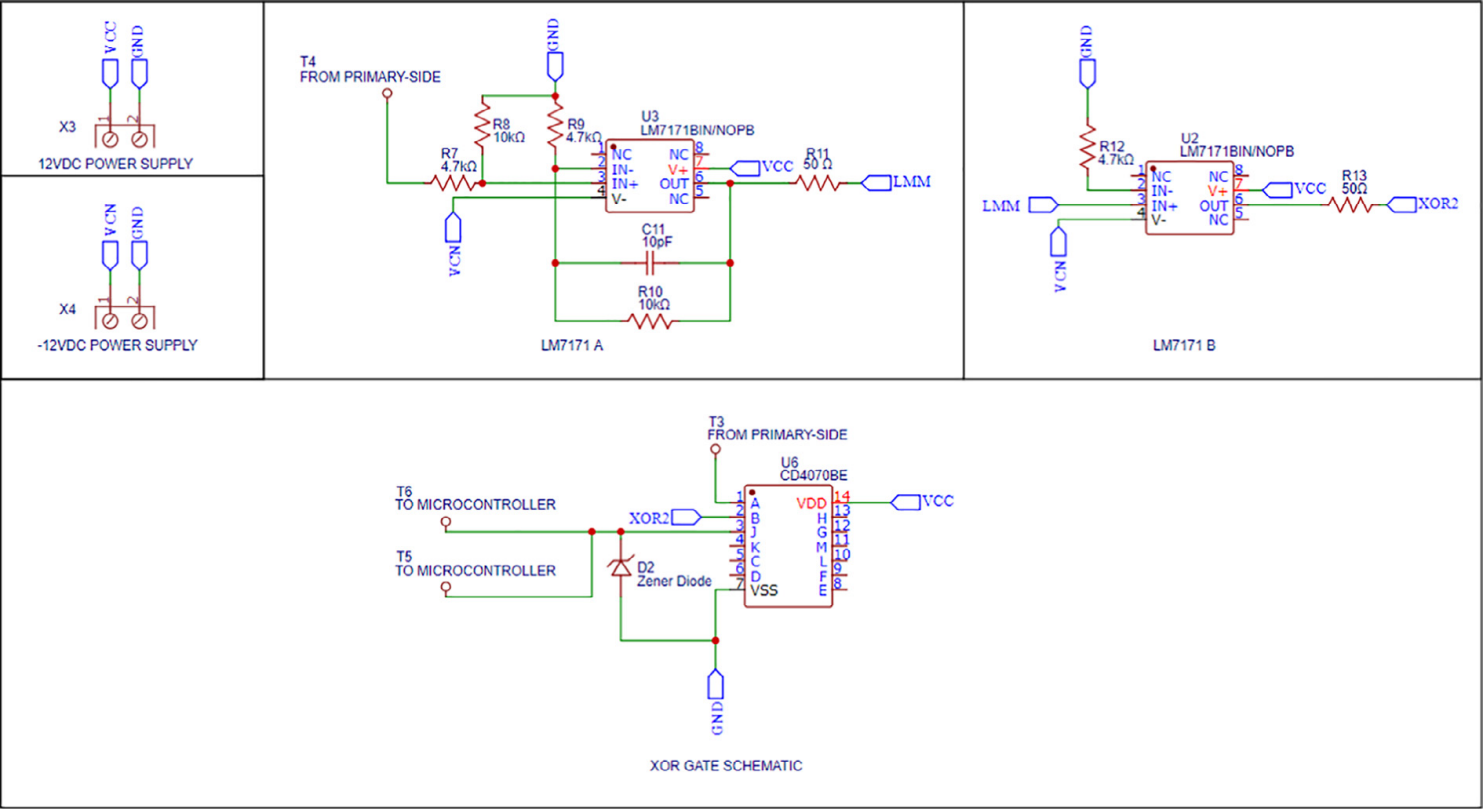
Phase shift measurement module’s wiring diagram.

**Fig. 12:**
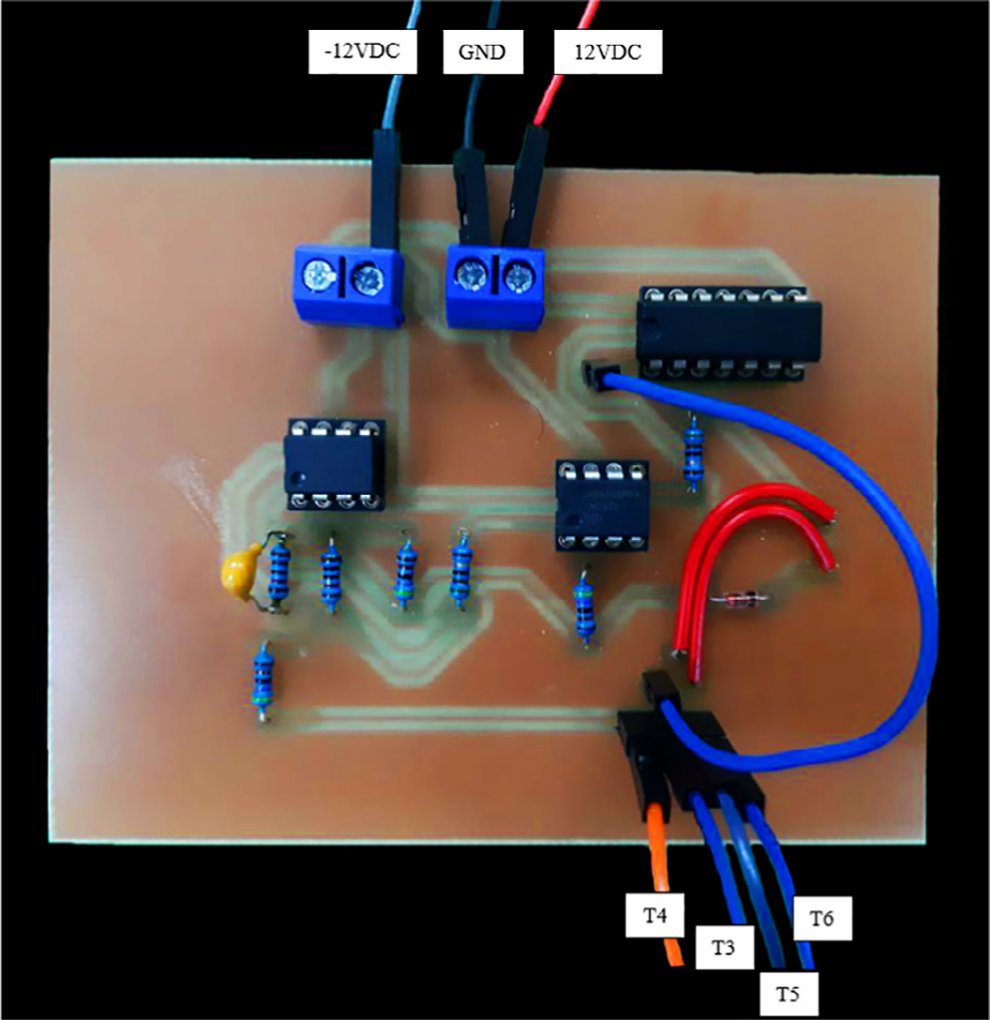
Phase shift measurement module’s PCB.

The phase shift measurement module’s wiring diagram is shown in Fig. 11 (available as well in Phase_shift_wiring_diagram.pdf). The PCB layout and the PCB manufacturing files are found in Secondary_side_PCB_layout.json and Phase_shift_PCB_manufacturing.zip, respectively. Fig. 12 shows the phase shift measurement module’s PCB assembly. Similarly to the previous section, follow the same instructions for manufacturing the PCB. The same safety concerns and recommendations are applied to this module.

### Control module manufacturing and programing

**Fig. 13:**
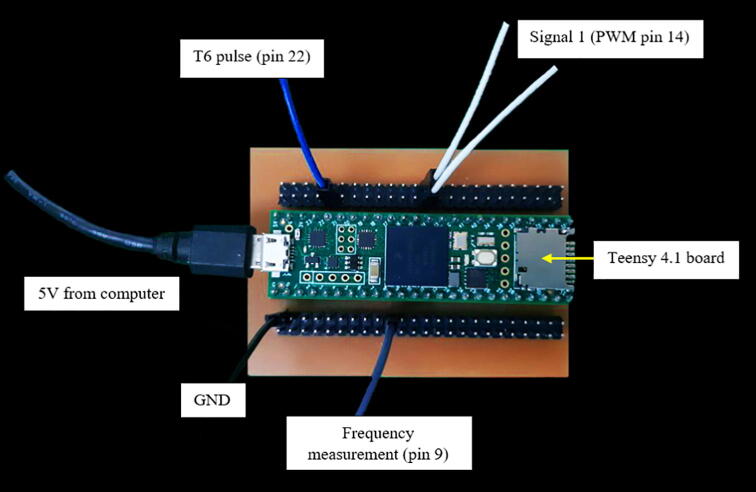
Control module’s PCB.

The control module’s PCB layout and PCB manufacturing files are found in Control_module_PCB_layout.json and Control_module_PCB_manufacturing.zip, respectively. Fig. 13 illustrates control module’s PCB assembly. Similarly to the previous section, follow the same instructions for manufacturing the PCB. The same safety concerns and recommendations are applied to this module.

The online repository contains the three INO files (see Table 2) that can be uploaded to the Teensy 4.1 board with VS code or Arduino IDE via micro-USB cable. The download of the MedianFilterLib2 is required for obtaining more accurate results with the microcontroller, regardless of the coding environment. For the Arduino IDE users, the only additional installation needed is the Teensyduino add-on. On the other hand, VS code users need to download and install all the following complements:

• Teensyduino add-on
• Arduino extension for VS code
• C/C++ extension for VS code
• VisualTeensy add-on
• TyTools add-on

Instructions for the installation and use of every complement are given in their respective online repository. Note that the advantage of VS code environment is that it offers more professional features for coding, especially when linking the programing with an online repository and working with several teammates. Otherwise, we recommend Arduino IDE.

The files PWM_signals_test.ino and Pulse_width_frequency.ino are meant to be used for testing purposes before the final implementation, which is the PI controller, available in the online repository shown in Table 2. The operation instructions section offers more details about the use of these files.

### 3D-printed displacement system manufacturing

**Fig. 14:**
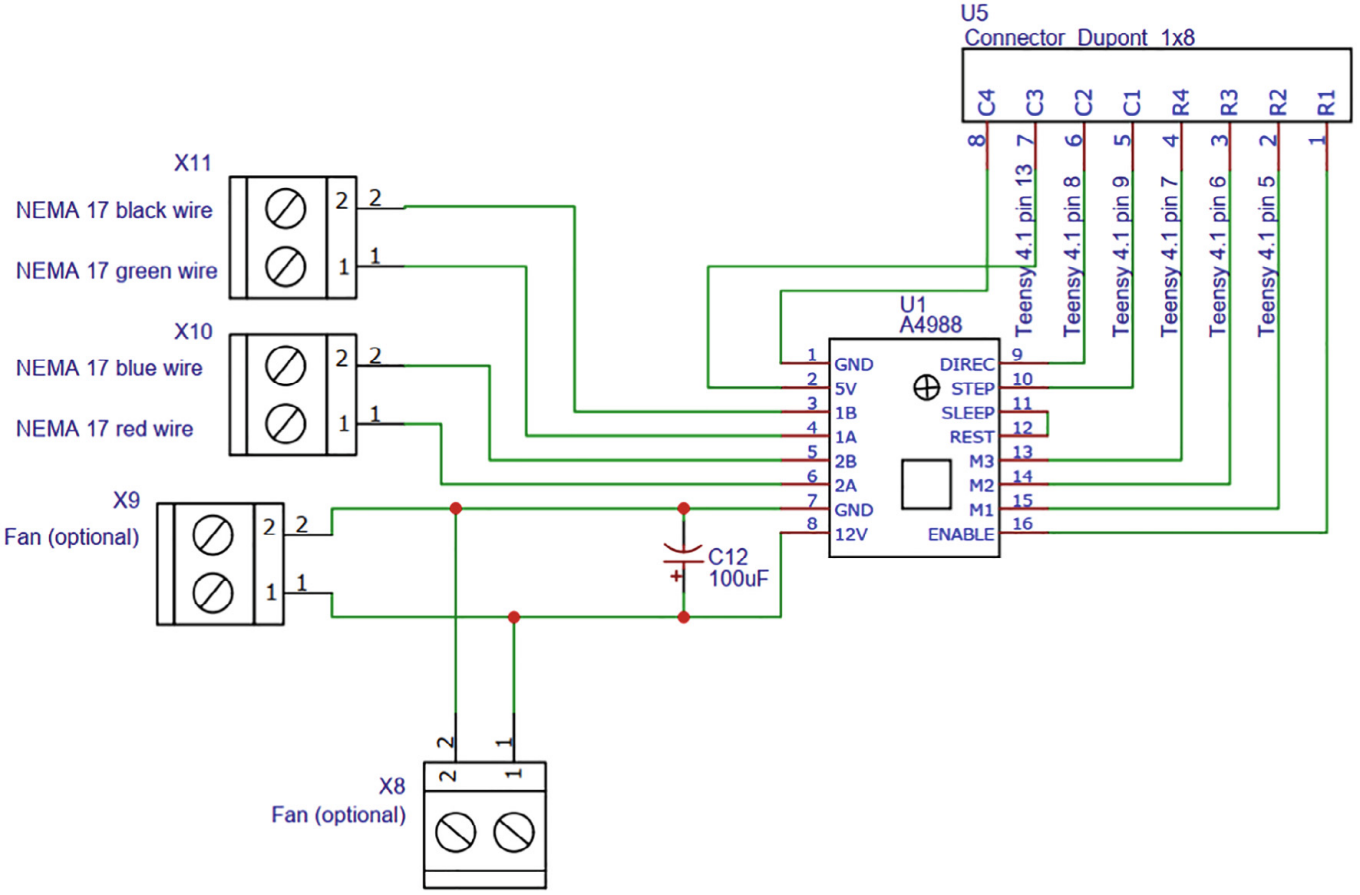
Displacement system’s wiring diagram.

The displacement system utilizes a stepper motor NEMA 17 (details provided in the BOM section) to produce the torque necessary to elevate the receiving coil vertically. For controlling the stepper motor, the A4988 stepper motor driver was selected to work along with a Teensy 4.1 microcontroller. The electrical connections are illustrated in Fig. 14. The circuit’s diagram can be directly opened with any design software by uploading the file DS_wiring_diagram.json. The PCB layout and the PCB manufacturing files are found in DS_PCB_layout.json and DS_PCB_manufacturing.zip, respectively. The same safety concerns and recommendations described in previous sections are applied to this module.

To build the acrylic structure, follow the directions below and make sure to use the file **DS_instructions_drawings.pdf**, as the main guide, as it contains step by step building instructions and details about every piece of the module.

• To 3D print the supports structures, load the STL files located in 3D_printable_support_structures.zip in a printing software (you can download and install the QIDI printing software in http://www.qd3dprinter.com/software/). Next, configure the preferences for every piece and save the file to a removable drive. Plug the drive to the printer, set up the printer and start printing.
• For the laser cutting, load the files located in Acrylics_laser_cutting.zip in a laser cutting software (Glowforge software is available in https://app.glowforge.com/). Carefully introduce the acrylics in the printer slot, close the lid and wait for the software to align the designs. Once the piece is aligned, start printing. When the printing is finished, open de lid and remove the acrylics slowly. For safety reasons, do not open the lid while the printer’s laser is still operating.
• Use the file **DS_instructions_drawings.pdf** as a guide to assembly all the pieces using the specified screws.
• Place the stepper motor, the displacement system PCB module, the Teensy 4.1 microcontroller and the receiving coil in the corresponding space.
• Proceed to read the operation instructions section.

**Fig. 15:**
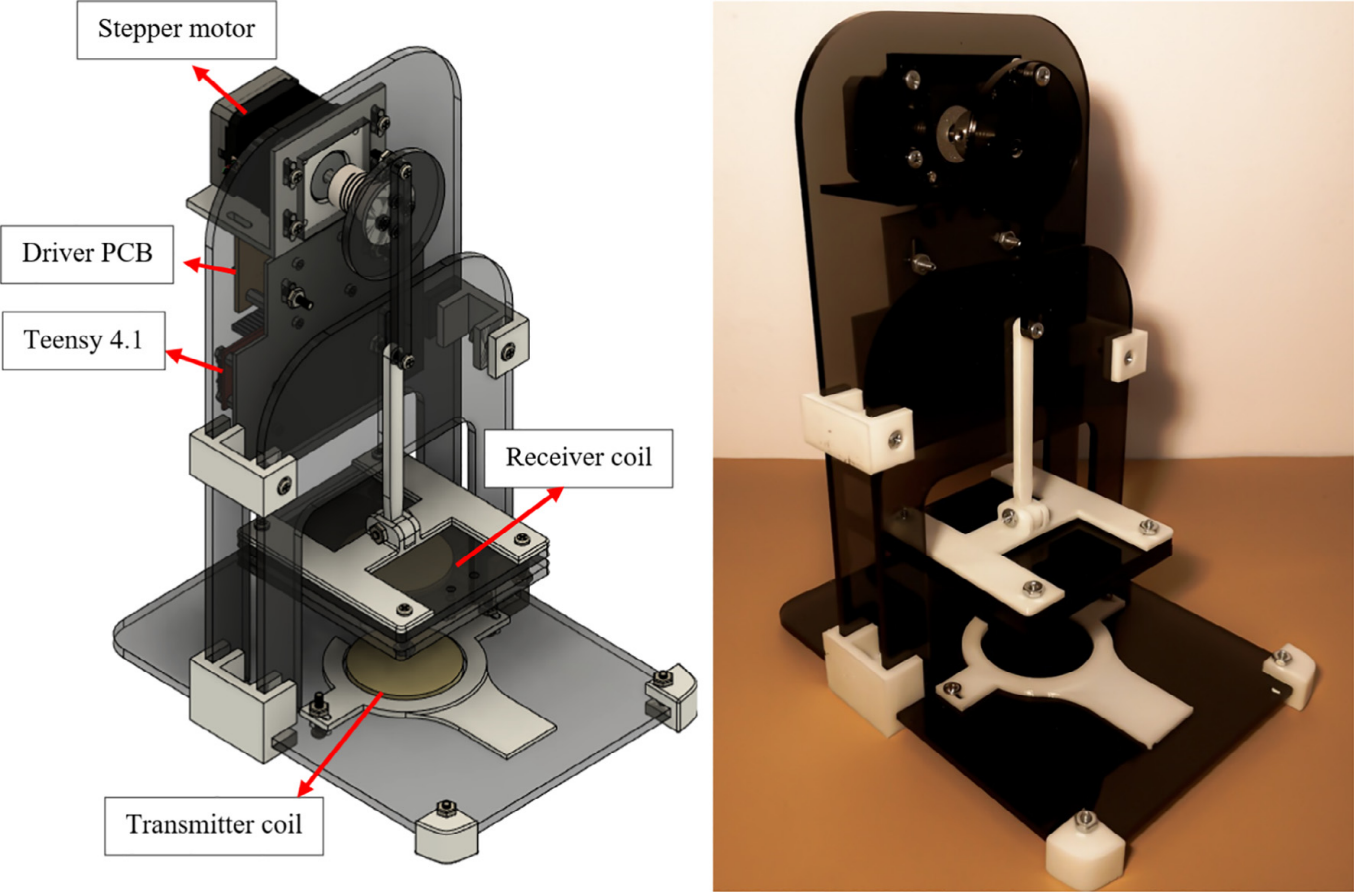
Displacement system final assembly (right: 3D model, left: real model).

The final assembly is illustrated in Fig. 15. Furthermore, the file DS_assembly_views.zip includes different views of the system (isometric view, top view, back view, front view). The Autodesk Fusion360 file is also available (DS_modifiable_3D_design.f3d). The coding and the LabVIEW interface will be explored in the operation instruction section below (section 6.2).

## Operation instructions

### Operation of the WPT modules

Before switching on the system, make sure that:

• The DC power supply is connected with the proper polarity in the primary side-module (Fig. 8) and the phase shift measurement module (Fig. 12).
• The desired microcontroller’s code is uploaded.
• The connections between the primary and the phase shift measurement module are correctly wired (Fig. 8 and Fig. 12). This also includes the connections between the control module and the two previously mentioned modules (Fig. 13).

Desired code selection:

• PWM_signals_test.ino: This code’s main function is to provide the primary-side module a desired PWM signal with a chosen frequency and duty cycle. It’s also a simple code that may be used to test the circuit’s functionality.
• Pulse_width_frequency.ino: Provides the same application as the forementioned but in addition it takes the signal that exits the XOR gate from the phase shift measurement module into the control module in order to measure the frequency and phase angle obtained. As stated before, this code is used only for testing purposes.
• pi_controller.ino: Combines the previous two codes and adds a PI controller which aids in finding the frequency which allows the system to provide the best possible power transfer between the primary-side and secondary-side modules regardless the positioning of these two.

Switching on the system:

First, the microcontroller provides a PWM control signal to the primary-side module. The frequency of this signal is selected by the operator, who is expected to select a frequency in the range specified in Table 1. Next, the phase shift measurement module generates a pulse that is proportional to the phase shift between waves in the transmitting antenna, which is read by a pin of the microcontroller. If the PI controller code is in use, then the microcontroller will automatically adjust the frequency in order to maximize the power transmission between the coils.

### Operation of the 3D printed displacement system

#### Arduino IDE code setup (same procedure for the VS code users but consider section 5.4)

1. Download and install Arduino IDE from: https://www.arduino.cc/en/software
2. Download and install Teensyduino from: https://www.pjrc.com/teensy/td_download.html
3. Copy and paste the “Libraries” folder from the repository to the Arduino IDE folder created after the installation.
4. Open Arduino IDE.
5. Open ‘‘Rot-Tras_Mechanism.ino” in Arduino IDE.
6. Connect Teensy 4.1 to the computer using a microUSB cable.
7. In Arduino IDE, navigate to Tools > Board: Select ‘‘Teensy 4.1″
8. Click ‘‘upload” and wait for the code to upload and wait for the ‘‘upload completed” message.

#### LabVIEW program setup

1. Access to the following website and select your PC specifications: https://www.ni.com/es-cr/support/downloads/software-products/download.LabVIEW.html
2. Download and install LabVIEW.
3. Extract the files from LabVIEW_interface.zip and download ResultsGen.vi.
4. Run LabVIEW and load ResultsGen.vi (the teensy must be connected and the code must be uploaded before running the application).

#### LabVIEW GUI description

**Fig. 16:**
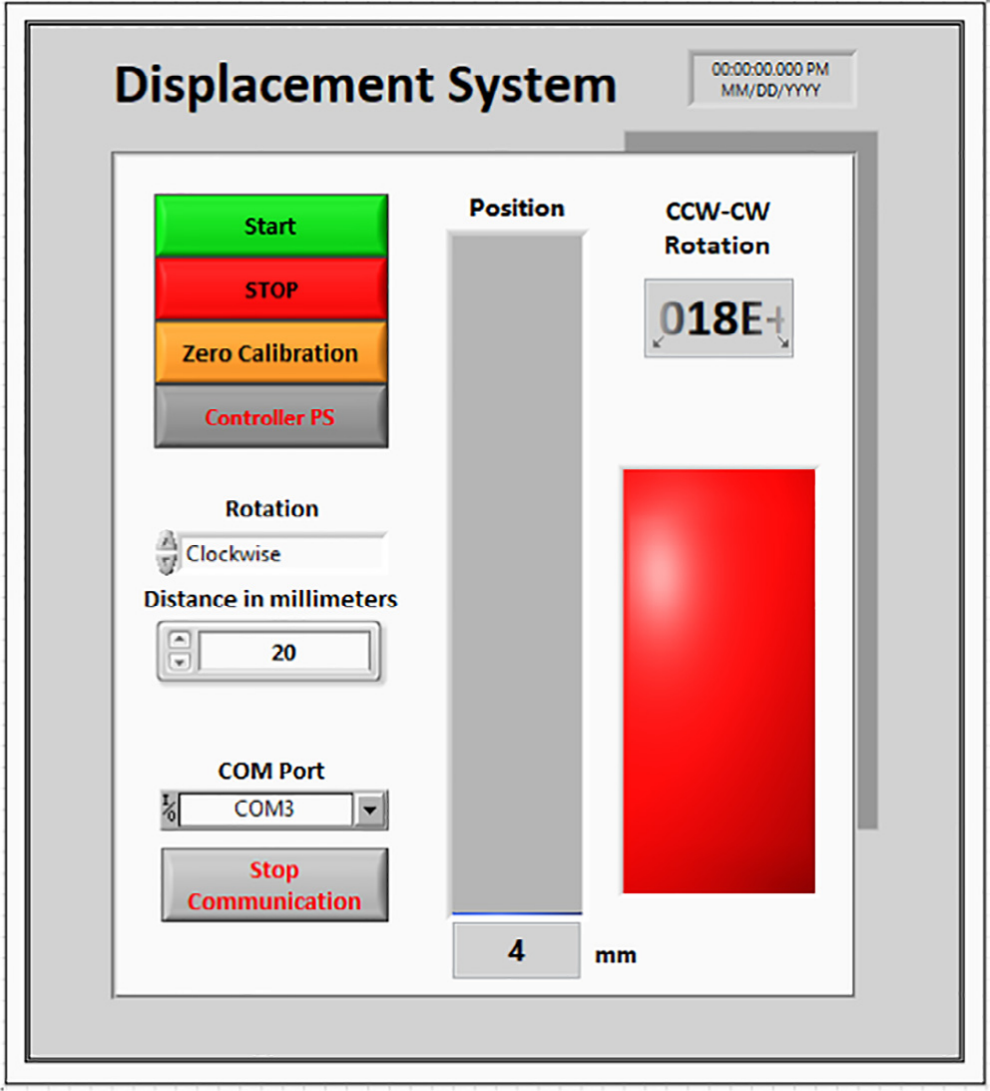
Displacement system graphical user interface.

The following descriptions all refer to Fig. 16.

• START: Initiates the displacement action
• STOP: Finishes the displacement action
• Zero Calibration: Sets the current position to as the starting position 0 mm.
• Controller PS (Power supply): Powers on the a4988 controller.
• Rotation: Lets the user decide the motor rotation between clockwise and counterclockwise
• Distance in millimeters: Sets the desired distance to be reached measured from the zero-calibration point.
• COM Port: Sets the desired serial communication port.
• Stop Communication: Terminates communication with the current COM Port.

The correct series of commands to operate the displacement system after setting up the Arduino program and LabVIEW is to:

1. Set the starting position with zero calibration.
2. Input the sense of the rotation of the motor.
3. Input the desired distance to displace the coil.
4. Select the COM Port that corresponds to the Teensy.
5. Turn on the controller.
6. Press start.
7. Set a new distance.
8. Adjust rotation sense if necessary and go back to step 6.

**Notes**.

• STOP is only used in case the user desires to terminate the displacement before it reaches the input distance.
• If the clockwise rotation was used to elevate the coil, then the counterclockwise must be used to lower it and vice versa.
• CCW is displayed as “0″ and CW as “1”.

## Validation and characterization

The validations and characterizations will be presented in different sections, according to the stages of the project.

### Load modelling

For the load modelling, the approach presented [1] was selected, where the authors used a constant resistance that represents the internal resistance of the battery that the system would charge. As a result, the charging mode of this prototype is constant resistance (CR). On the other hand, since the recommended operating frequency for WPT systems ranges between 79 and 90 kHz, and due to the size difference between our coils and the coils implemented in [1], our selected resistance values were different as well. In the present case, resistors of 1000, 500 and 100 O served as load models, in order to maintain the operating frequency between the recommended range.

Furthermore, in case an actual battery is to be used as a load, and a constant current (CC) or constant voltage (CV) charging mode is required, then the MH-CD42 module is needed to provide the appropriate protections and ratings to the battery.

### Bode plot of the system

**Fig. 17:**
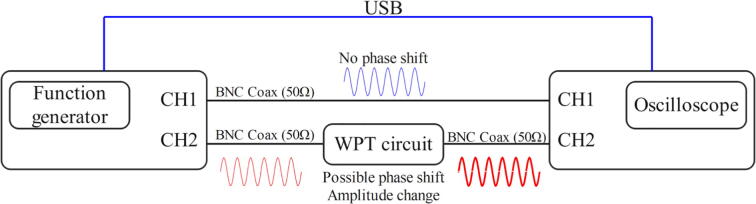
Wiring diagram for the Bode plot test.

**Fig. 18:**
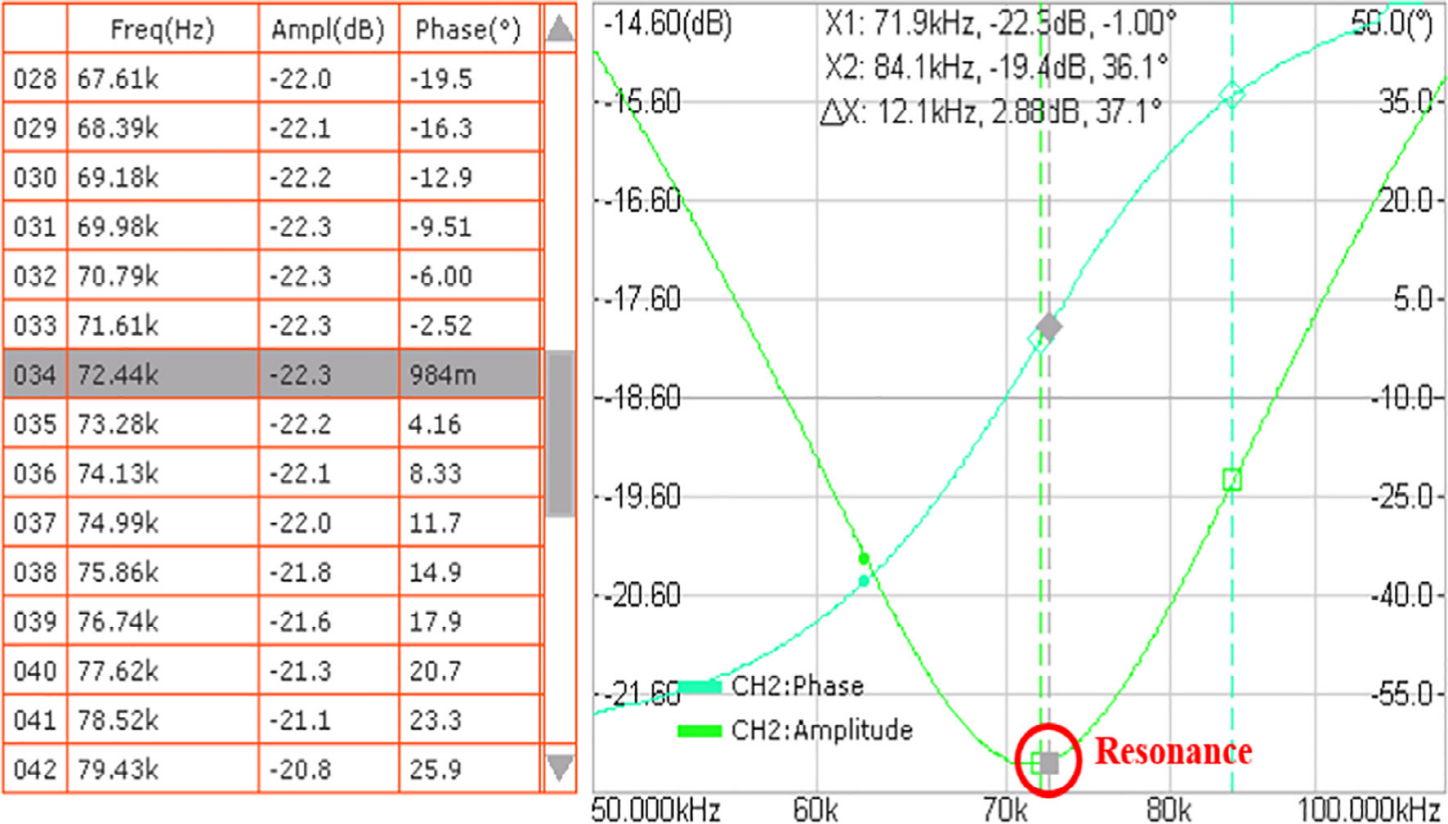
Bode plot of the system with the resonance frequency circled in red. (For interpretation of the references to colour in this figure legend, the reader is referred to the web version of this article.)

The Bode plot (i.e., the frequency response plot) allowed us to find the resonance frequency of the WPT prototype graphically, using a four-channel oscilloscope (model SIGLENT SDS1104X-E) and a two-channel function generator (model SIGLENT SDG1032X). The first step of this characterization is to open the oscilloscope’s Bode plot interface by pressing the ‘Utility’ button and then selecting the option ‘Bode Plot’. Secondly, it is necessary to set the range of the frequency sweep from 50 kHz to 100 kHz and the voltage amplitude to 2 V peak-to-peak. Subsequently, connect the function generator with the oscilloscope and the primary and secondary-side modules (Fig. 17). Since both CH1 and CH2 of the function generator need to output the same signal, press the ‘Utility’ button and enable the channel tracking mode. Once this assembly is complete, turn on CH1 and CH2 of both devices as well as the ‘Operation’ mode in the Bode plot interface. The oscilloscope should start plotting the amplitude and the phase angle (of the voltage) through the range of frequencies. To pinpoint the precise resonance frequency, locate the point where the phase angle is zero, which should coincide with the minimum amplitude in decibels. As seen in Fig. 18, a very close point corresponds to a frequency of 72.44 kHz, which is quite similar to the theoretical resonance frequency calculated in the primary-side module description.

### Voltage, current and pulse waveforms

**Fig. 19:**
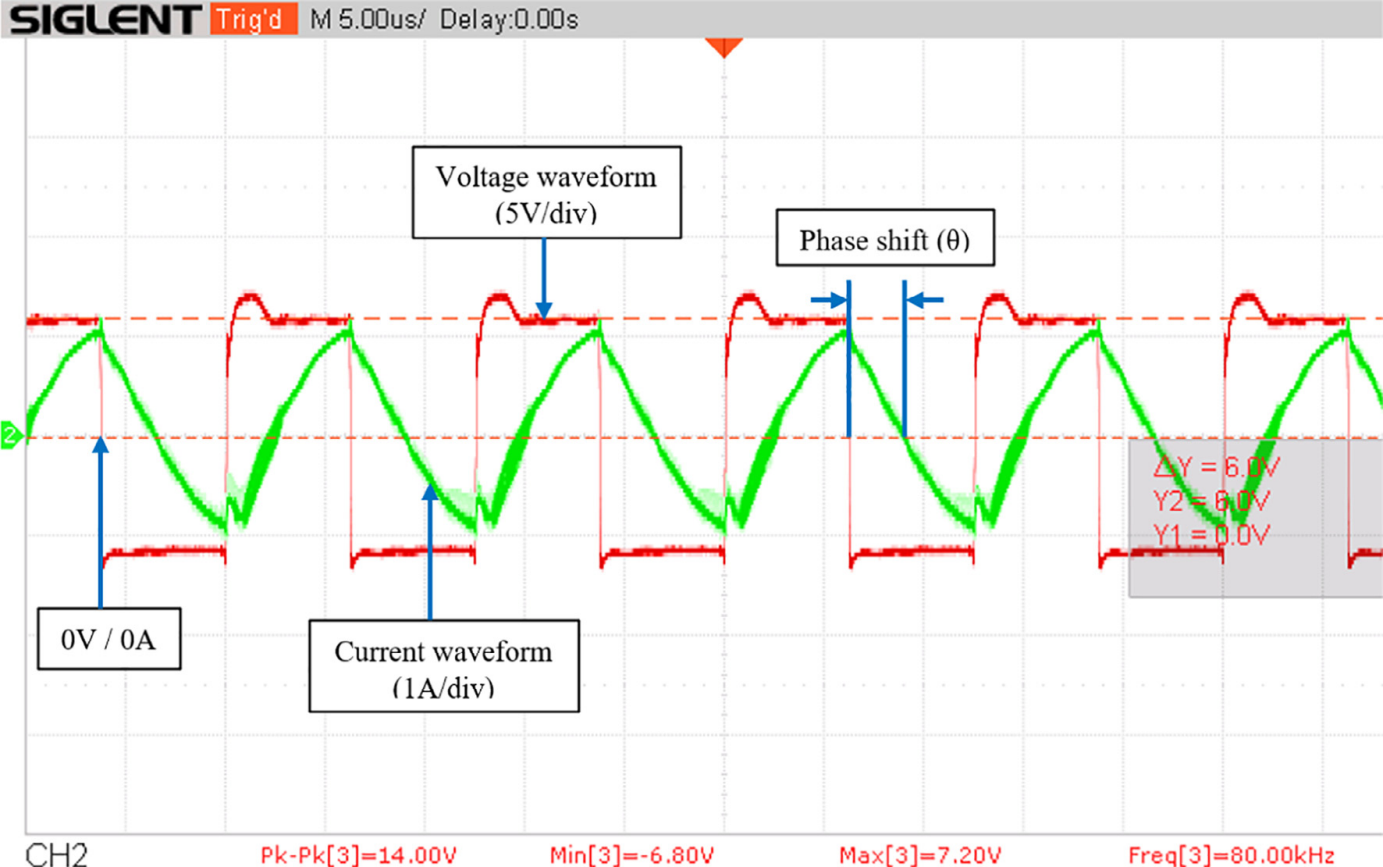
Voltage and current waveforms in the transmitting antenna at 80 kHz. The phase shift represents the time difference between the zero-crossing of the voltage and current waveforms.

Fig. 19 shows the voltage and current waveforms in the transmitting antenna with the system operating at 80 kHz. The voltage is a square wave from 6 V to −6V and the current has a sinusoidal behavior, as expected. Note that there is a phase shift between these two waveforms since the system has not been optimized yet. As explained in section 2.3, the phase shift is visualized as the time difference between the zero-crossings of the waveforms (i.e., the intersection with the 0 V/0A axis, where the sign of the functions changes). This occurs twice during a single cycle; therefore, the frequency of the phase shift pulse is twice the frequency of the voltage and current waveforms.

**Fig. 20:**
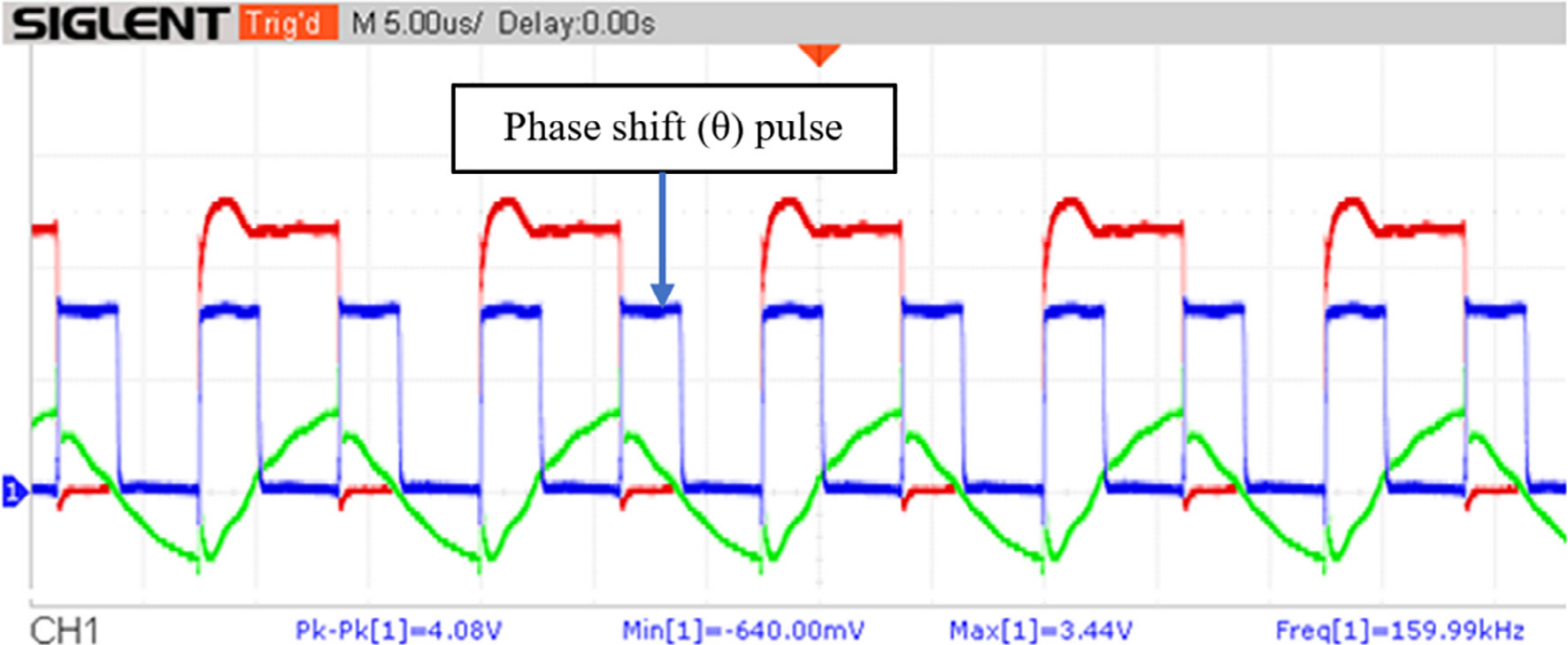
Phase shift represented as a blue voltage pulse with a 160 kHz frequency. The phase shift represents the time difference between the zero-crossing of the voltage and current waveforms. (For interpretation of the references to colour in this figure legend, the reader is referred to the web version of this article.)

On the other hand, Fig. 20 illustrates the phase shift measurement module’s output as a blue pulse that matches the corresponding phase shift. Note that the pulse’s frequency is 160 kHz whereas the voltage and current’s frequency is 80 kHz. Furthermore, it was corroborated that as the operating frequency approached the resonance frequency, the pulse’s width decreased until it disappeared, and the phase angle computed by the Teensy 4.1 approached zero. Once the value of the resonant frequency was exceeded, the pulse’s width increased its thickness again, indicating that the phase shift between the voltage and current waves also increased, as also confirmed by the phase angle calculated by the Teensy 4.1.

### Teensy’s measurement display

**Fig. 21:**
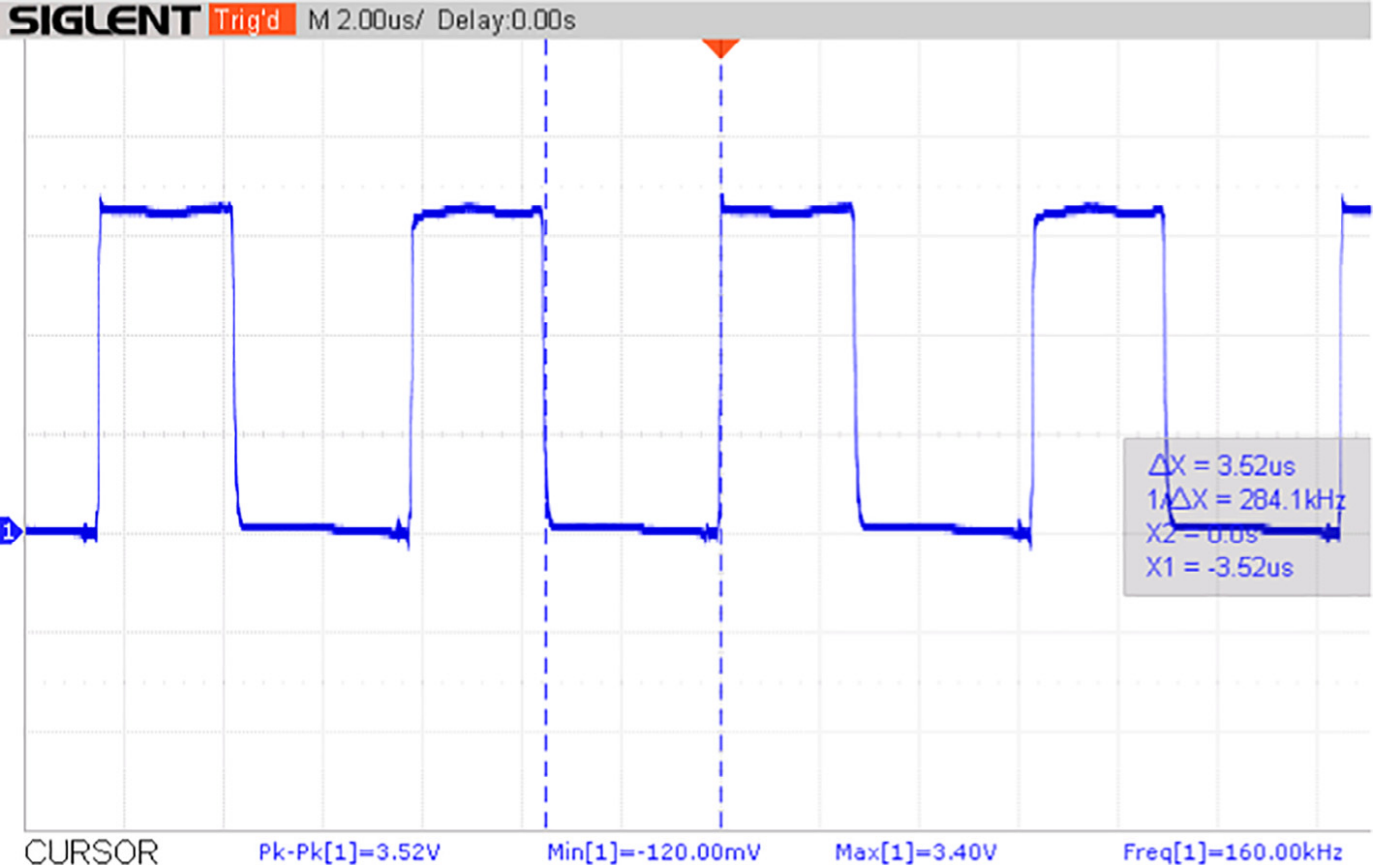
Voltage pulse’s off time at 80 kHz input (1 V/div).

**Fig. 22:**
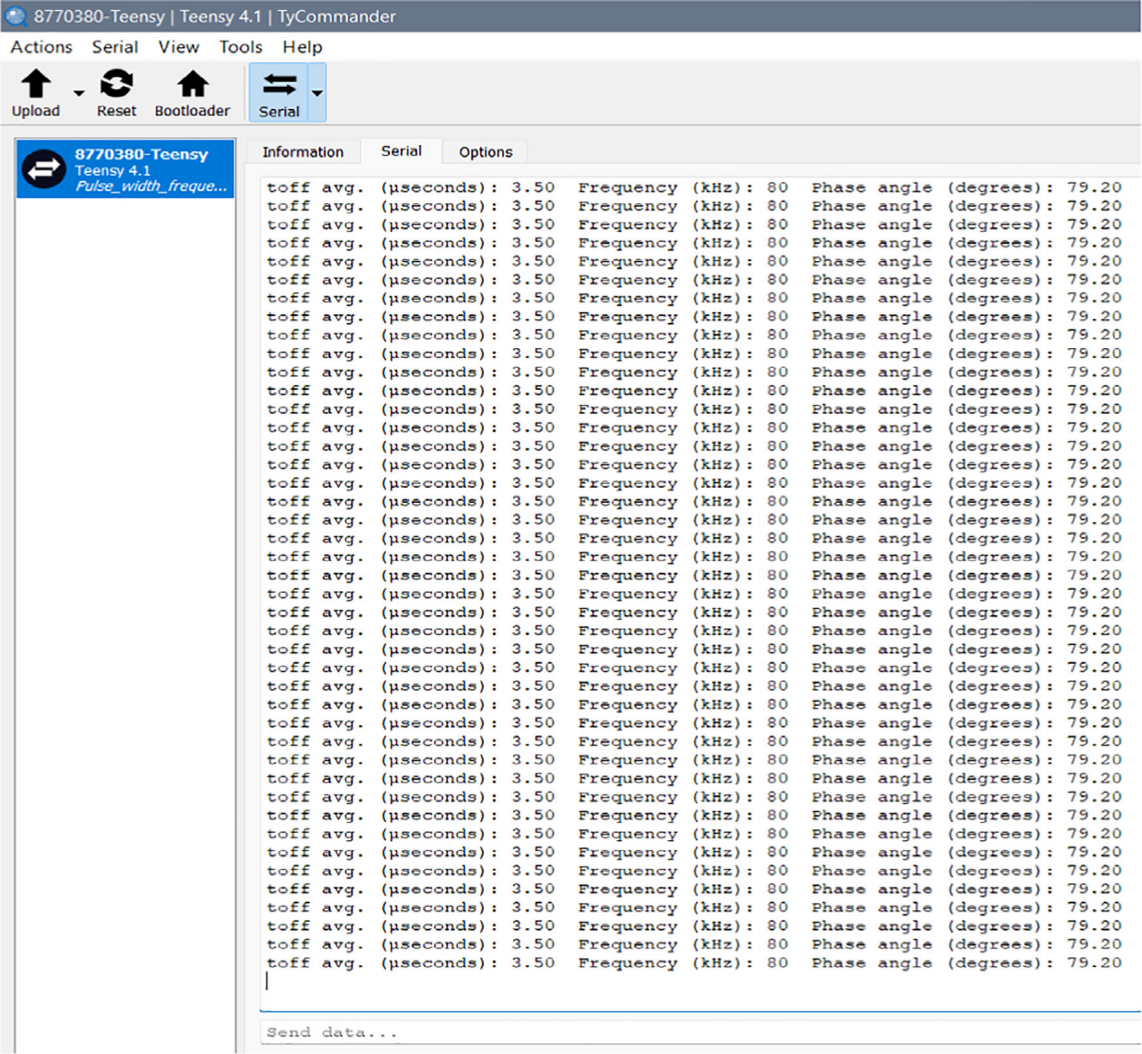
Serial monitor’s display with the average *off time*, the input frequency and the phase shift (80 kHz).

Using the Teensy 4.1 board and the TyTools add-on in VS code, it was possible to compute and display the phase shift between the voltage and current waveforms by reading *off time* and frequency of the pulse obtained from the XOR gate’s output (Fig. 21), as explained in the hardware description section. Using the display’s cursors, we can establish that the pulse has an *off time* of 3.52 microseconds at an 80 kHz input (160 kHz output). In addition, Fig. 22 shows the serial monitor’s display with the average *off time* (3.50 microseconds, which corresponds to a 0.57% error), the input frequency (80 kHz, half the pulse’s frequency) and the phase angle (79.2°) computed by the microcontroller’s algorithm.

Additionally, the following behavior was corroborated: if we operate the system at a frequency closer to the resonance frequency, the width of the voltage pulse diminishes until almost disappearing and the *off time* is maximized.

### Optimization via PI controller

**Fig. 23:**
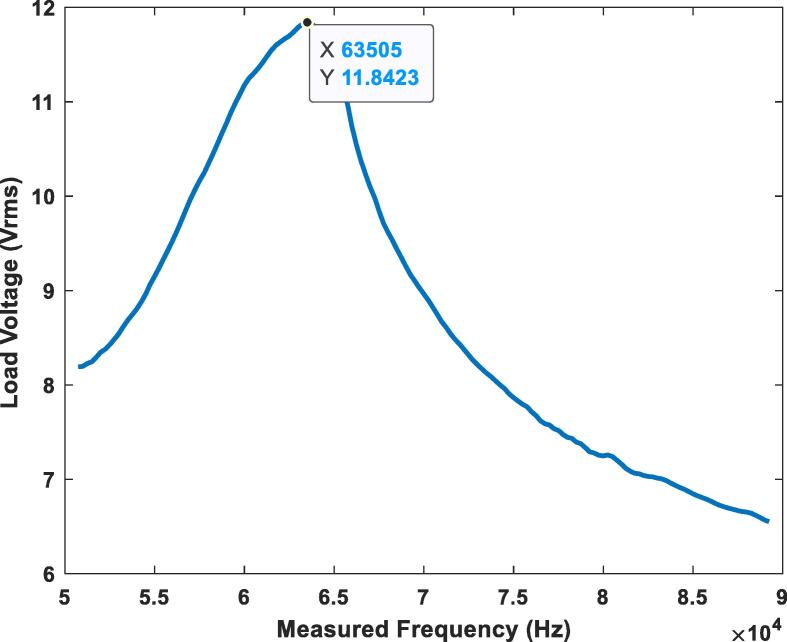
Frequency sweep plot for a 100-ohm load at zero misalignment between the coils.

Once it was demonstrated that the Teensy 4.1 board can furnish reliable calculations for the phase angle, the PI controller implementation was performed. In this characterization, a series of vertical misalignments were introduced between the two antennas using the 3D printed displacement system, these being z0 = 0 mm, z1 = 4 mm, and z2 = 8 mm. After each corresponding vertical misalignment was set, the PI controller algorithm was uploaded to Teensy 4.1 board to optimize the system. This procedure was implemented for three different load conditions: a 100-ohm resistor, a 500-ohm resistor, and a 1000-ohm resistor. To begin with, another frequency sweep was performed, but this plot (Fig. 23) exhibits the load voltage behavior in RMS values, obtained with the acquisition and analysis of data stored with the acquisition system described in section 7.6.

Fig. 23 describes the behavior of the voltage when the frequency approaches the resonance point during a sweep from 50 kHz to 90 kHz and a load of 100-ohms with no misalignment (z = 0 mm). In this case, at a frequency of 63.5 kHz, a maximum voltage of 11.8 Vrms was obtained. The voltage transmitted to the load increases until the resonance frequency is obtained. As soon as the frequency surpasses the resonance point the voltage starts diminishing.

#### Optimization results with a 100-ohm load.

**Fig. 24:**
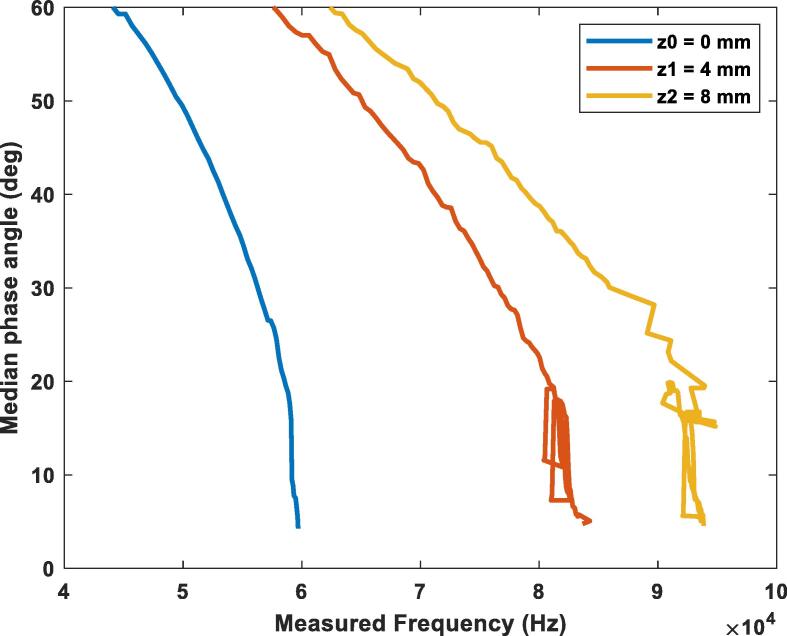
Phase angle variation while using the PI controller for a load of 100-ohms at different vertical misalignments.

Fig. 24 shows the change of the phase angle with the frequency. The test was performed with a 100-ohm resistor at three different vertical misalignments: z0 = 0 mm (blue), z1 = 4 mm (orange), z2 = 8 mm (yellow). It’s seen that as the gap between the coils increases the device must produce higher frequencies to achieve the resonance point. In this case the resonance frequency went from 60 kHz at a 0 mm gap to a 93 kHz frequency at an 8 mm gap with the 100-ohm load. The setpoint of the control algorithm was set to a threshold of less than 5 degrees of phase-shifting.

#### Optimization results with a 500-ohm load.

**Fig. 25:**
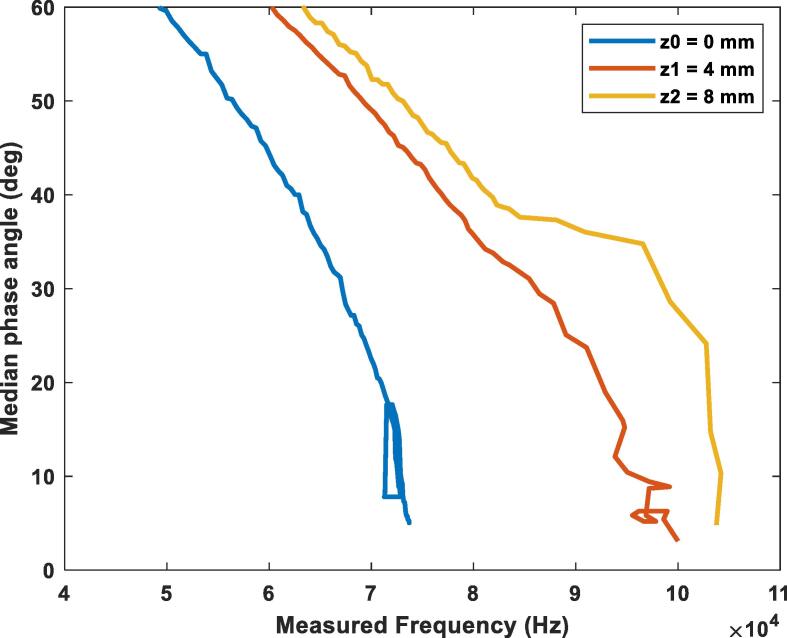
Phase angle variation while using the PI controller for a load of 500-ohms at different vertical misalignments.

Fig. 25 shows the change of the phase angle with the frequency. The test was performed with a 500-ohm resistor at three different vertical misalignments: z0 = 0 mm (blue), z1 = 4 mm (orange), z2 = 8 mm (yellow). As observed from before, as the gap between the coils increases the device must produce higher frequencies to achieve the resonance point. The main takeaway when compared with a lower load as the one of the graphs before is that due to this load being larger, the resonance frequency in each height is slightly higher as well. The setpoint of the control algorithm was set to a threshold of less than 5 degrees of phase-shifting.

#### Optimization results with a 1000-ohm load.

**Fig. 26:**
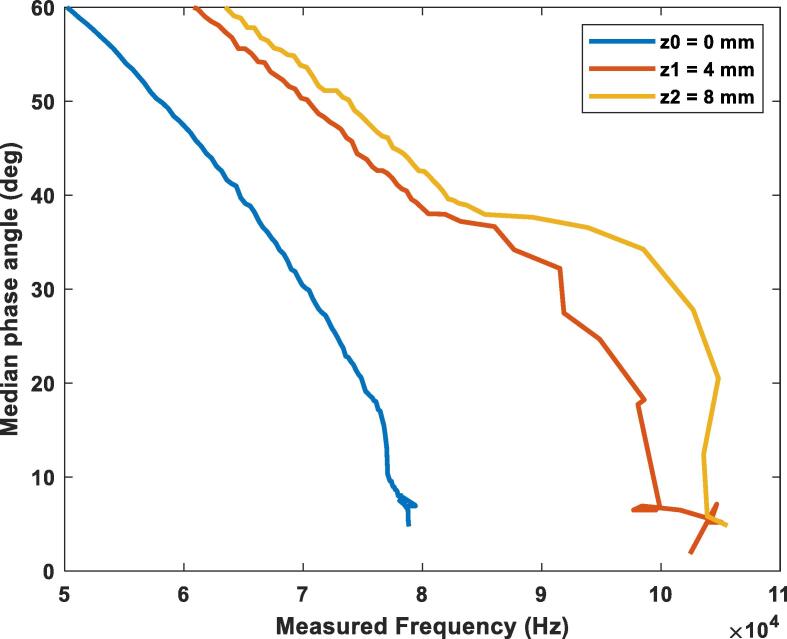
Phase angle variation while using the PI controller for a load of 1000-ohms at different vertical misalignments.

Fig. 26 shows the change of the phase angle with the frequency. The test was performed with a 1000-ohm resistor at three different vertical misalignments: z0 = 0 mm (blue), z1 = 4 mm (orange), z2 = 8 mm (yellow). This case continues to present the before seen behavior of the increase in resonance frequency as the height between the antennas increases. Just as seen in the previous graph, the larger load made the resonance frequency increase even more for all the heights tested.

It can be concluded that the controller accomplished its main task, which was optimizing the operation by finding the resonance frequency under a variety of the misalignments and load conditions. Better results could be obtained with a lower threshold for the PI controller, but special attention should be current could increase.

### Data acquisition system

The data were acquired using a LabVIEW interface that collected the information from the outputs of the devices measuring the phase-shift behavior and the voltage response of the wireless power transfer prototype via USB connections. Specifically, the interface stored the frequency and the phase shift computed by the Teensy 4.1 microcontroller after each iteration of the PI controller, and similarly, stored the phase shift pulse width (necessary for calculations) and load voltage, which were captured by the oscilloscope’s channels. In addition, the current of the controllable power supply that feeds the system was recorded. Subsequently, the captured data were organized, filtered and analyzed using Matlab. **The complete experimental data on the performance of the WPT device on these laboratory test can be found in** https://data.mendeley.com/datasets/74ptpjsd5z/1.

**Fig. 27:**
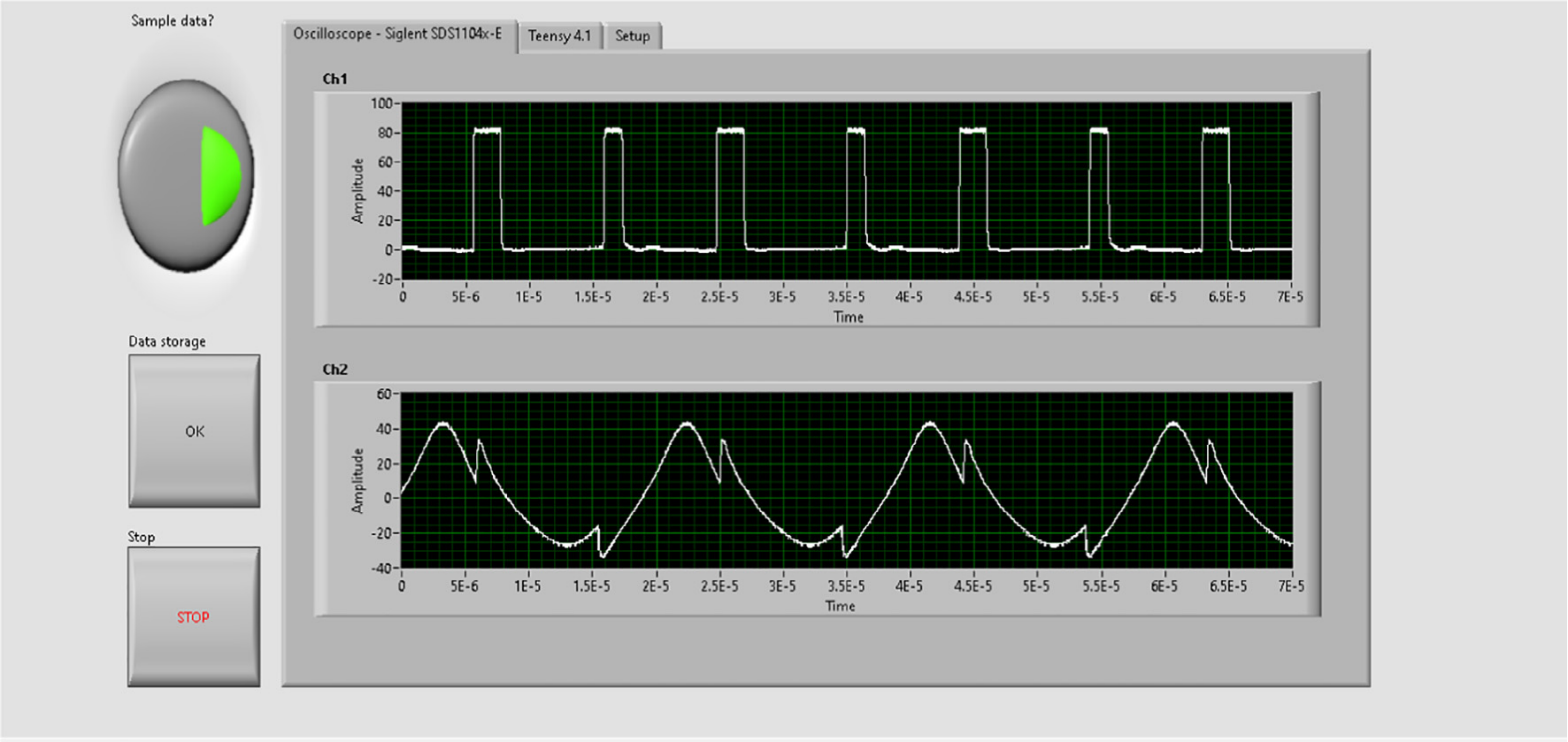
LabVIEW interface’s first tab containing channel 1 and 2 of the oscilloscope.

**Fig. 28:**
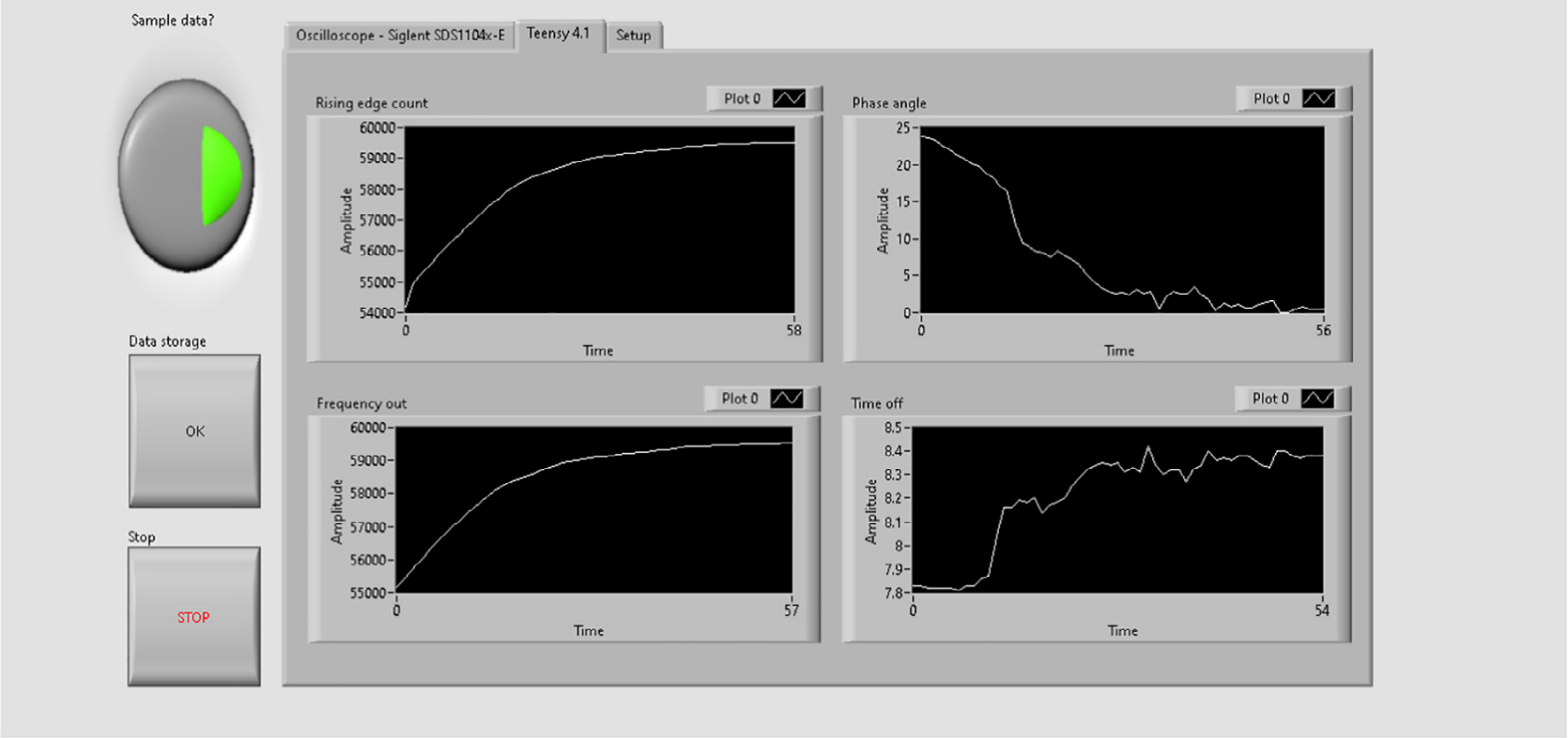
LabVIEW interface’s second tab containing the data acquired and processed by the Teensy 4.1.

**Fig. 29:**
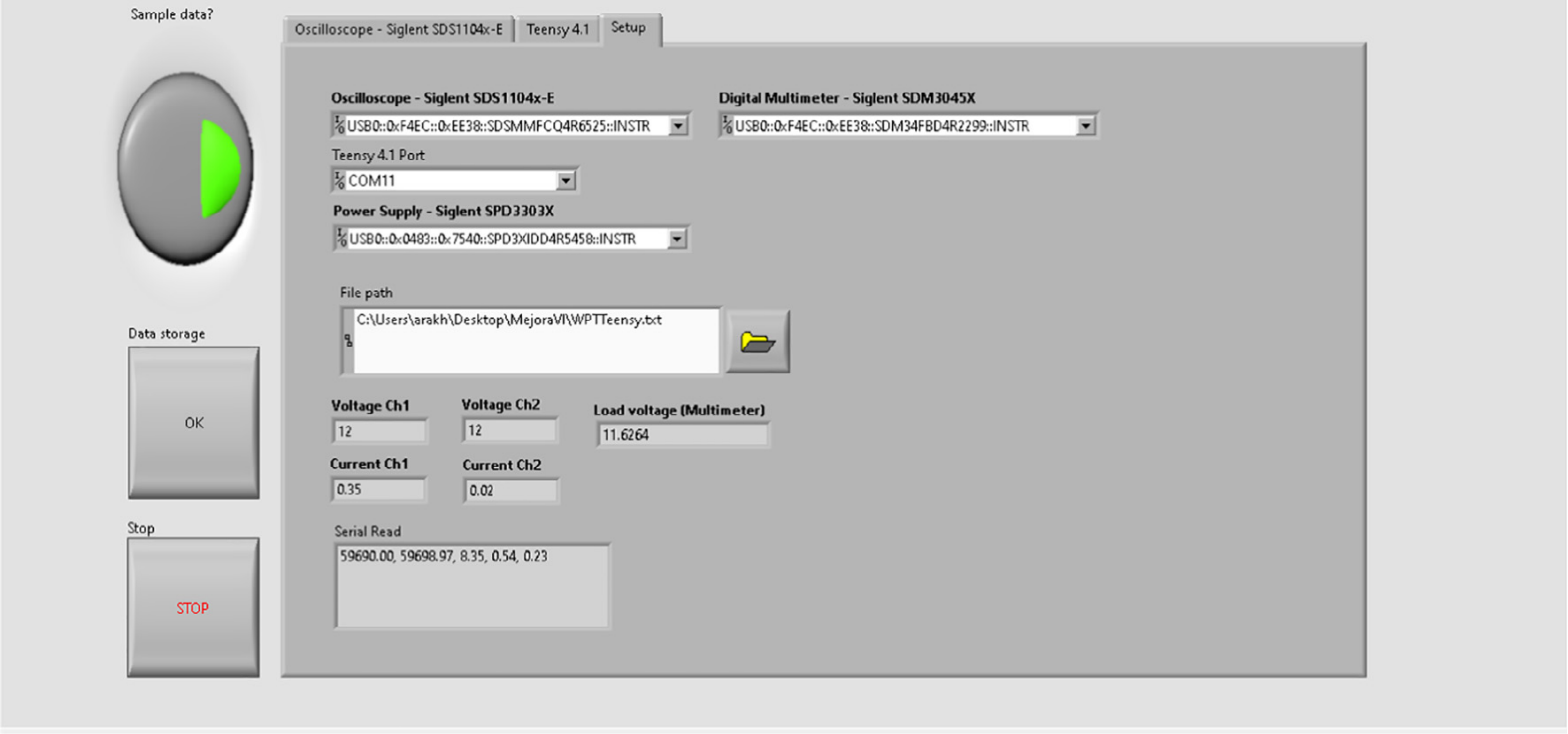
LabVIEW interface’s third tab used to setup the SIGLENT devices, the Teensy 4.1 port and file path.

The algorithm processes the data at a rate of 1 data per second, this is limited by the speed of data acquisition from the oscilloscope. The independent system (the device without the oscilloscope connected) takes an “n” amount of cycles times the time in each iteration, here each iteration had an approximate time of 12 microseconds. On the other hand, the Teensy 4.1 microcontroller presents a limitation which consists in that it’s only able to assign frequencies which are multiples of the internal system clock to the pins. This causes an error that overwrites itself from iteration to iteration and it cannot be fixed unless the Teensy is reset. This reset made every time the program starts, and synchronization of data between the oscilloscope and the Teensy as well as the parameters in the power supply and oscilloscope were captured and controlled by the LabVIEW interface shown in Fig. 27, Fig. 28, and Fig. 29. From Fig. 27, the pulse width can be observed in the oscilloscope’s channel 1. Channel 2 the sinusoidal current in the primary side of the prototype.

## CRediT authorship contribution statement

**Andrés Martínez:** Conceptualization, Methodology, Software, Validation, Investigation, Writing – original draft, Writing – review & editing, Visualization. **Christian González:** Conceptualization, Methodology, Software, Validation, Investigation, Writing – original draft, Writing – review & editing, Visualization. **Adrián Jaramillo:** Methodology, Software, Resources, Validation, Investigation. **Dorindo Cárdenas:** Conceptualization, Methodology, Supervision, Writing – review & editing. **Alejandro Von Chong:** Conceptualization, Methodology, Software, Validation, Supervision, Investigation, Resources, Writing – review & editing, Project administration.

## Declaration of Competing Interest

The authors declare that they have no known competing financial interests or personal relationships that could have appeared to influence the work reported in this paper.
